# Engaging with change: Information and communication technology professionals’ perspectives on change in the context of the ‘Brexit’ vote

**DOI:** 10.1371/journal.pone.0186452

**Published:** 2017-11-08

**Authors:** Elizabeth Lomas, Julie McLeod

**Affiliations:** 1 iSchool, Department of Information Studies, University College London, London, United Kingdom; 2 iSchool, Department of Computer and Information Sciences, Northumbria University, Newcastle-upon-Tyne, United Kingdom; University of Texas at San Antonio, UNITED STATES

## Abstract

**Background:**

Information and Communication Technology (ICT) has been a key agent of change in the 21^st^ century. Given the role of ICT in changing society this research sought to explore the responses and attitudes to change from ICT professionals and ICT academics in dealing with the potentially far reaching political challenge triggered by the UK’s 2016 European Union Referendum and its decision to leave the European Union (referred to as Brexit). Whilst the vote was a UK based decision its ramifications have global implications and as such the research was not confined to the UK.

**Methods and findings:**

Data was collected through a survey launched on the first working day after the Brexit referendum vote to leave the EU and kept open for four weeks. The survey contained qualitative and quantitative questions. It sought to understand the opportunities and threats that would exist post-Brexit for ICT professionals and academics triggered by the decision. The research captured a complex rich picture on ICT professionals’ responses to the potential challenge of change triggered by the Brexit vote. Immediately after the Brexit decision the research reveals uncertainties amongst ICT professionals regarding what the decision would mean, with just under half of the participants not identifying any opportunities or threats. For those who did, threats outweighed opportunities by just more than double. Whilst understanding the global possibilities and dangers, participants saw their position from national and organizational perspectives. The highest frequency coded threats related to areas outside the participants’ control and the highest frequency opportunities related to areas where there was the potential for ICT interventions. This survey is part of longitudinal piece of research. Using the same methodological approach two further surveys are planned. The second survey will be one year after Article 50 was triggered on 29 March 2017. The final survey will be one year after the UK exit from the EU, assuming this occurs.

## Introduction

Information and Communication Technology (ICT) has been a key agent of change in the 21^st^ century. Over the last 20 years the rapid evolution of ICT has resulted in the development of complex information networks which cross political, legislative and geographic boundaries transforming communication and trading models. The economic, social and cultural capital of both the information which resides in ICT systems and the systems per se, in terms of software and hardware production, have increasingly been measured and included within GDP statistics [1]. Given the role of ICT in driving societal change, this research sought to explore perceptions of the opportunities and threats for ICT professionals in the light of possible changes triggered by the challenge of the UK referendum vote to leave the European Union (EU), colloquially referred to as ‘Brexit’ [2]. The intention was to capture a rich picture focusing on their perceptions of the opportunities and threats post-Brexit for them and their organizations, and the role that ICT can play in underpinning transformation.

This article first provides background on the role of ICT in the context of global change, followed by a brief overview of the EU and a summary of the events leading to the Brexit referendum. It then discusses the findings of a global survey which sought to capture ICT professionals’ responses to challenge and change using the Brexit referendum decision as the trigger for change. The research provides some insight more generally on the nature of national change and its wider global impact. The data also serves as a benchmark for a longitudinal study which will collect data at further critical points in the Brexit process.

## The role of ICT in global change

The concept of new machinery driving economic and social change is not new and has been discussed by philosophers and political commentators from Aristotle (in terms of war machinery) to Karl Marx (in terms of manufacturing machinery) to Albert Borgmann [3] today (in terms of information and technology). In this context Mutekwe [4] describes four key ages of society from domestication to agriculture, industry and finally the current information society. In the latter context, the idea of information access and understanding is linked to the changes in technology channels through which it flows. As noted by Dearnley and Feather [5] “The internet is not confined by the traditional boundaries of nations, races or classes…. It allows virtually instantaneous global communication of data of all kinds, and for that reason underpins the phenomenon of globalisation” (p. 51). Connecting and sharing information has enabled changes in wide ranging ways across society, with mixed impacts and outcomes.

Technological change has played a leading role in global transformation, impacting on the economy, trade, warfare, political mobilization, and identity formation [6]. It has challenged traditional media and political networks as people have been able to access and share new sources of information [7, 8]. The capacity of social media channels, the Internet of Things, cloud computing, blockchain, drones and artificial intelligence are changing the world in which we live. In addition, ICT has been claimed to operate across and even transcend borders and legislative regimes [9, 10]. System networks can be structured to allow data flow and storage to reside in multiple regimes simultaneously. The delivery is driven and supported by experts who are used to engaging with and supporting change.

Global networks have shifted business models in terms of global supply chains for goods and services [11]. In 2015, ICT products were estimated to account for 6% of the world’s global economy [12] excluding valuations for the goods and services which would not otherwise be supplied without the infrastructure provided by ICT. The impact and value of this ICT industry is growing [13], creating jobs and providing key infrastructures which sustain society.

Within this context, the UK is a significant economic market and supplier for the ICT industry. In 2015, it was calculated that the UK’s digital tech industry turned over an estimated £170 billion [14]. The UK provides the largest mobile device market in Europe [15]. About 207,000 ICT businesses operate in the UK, many of which are global entities including BT, Google, Hewlett-Packard and IBM. [16]

In a globally connected world, the decisions of one nation can have wider international reach through the impact of change on a chain of international agreements, laws and the operating environments for society and global corporations. For example, in the wake of the Lehman Brothers’ collapse in September 2008, there was a significant economic impact upon nations across the globe with *The Guardian* reporting that this caused a number of countries, including Hungary, Iceland, Latvia, Lithuania and Pakistan, to be caught in an economic downward spiral [17]. The UK’s Brexit decision to leave the EU in 2016 is one such decision with potentially significant implications that extend beyond the UK to other countries with whom it is networked.

The immediate ‘aftermath’ of the referendum result provided a context in which to begin a longitudinal exploration of the perceptions of ICT professionals, delivering information agendas in any sector, about the opportunities and threats presented by Brexit, and the role that ICT could play in underpinning this transformation.

## The European Union and Brexit context

### Development of the European Union in brief

The origins of the European Union (EU) lie in the 1951 Treaty of Paris and a 1965 treaty that created the European Communities, comprising six European countries that did not include the UK. The late 1960s saw the removal of import duties between the six members, and the 1970s saw the introduction of the Exchange Rate Mechanism. The Single European Act was signed in 1986 to create a single market by the end of 1992, and in 1992 the Maastricht Treaty was signed. This created the European Union, established the foundations to enable a single European currency (the Euro), and provided the basis for wider cooperation on foreign and security policy. Further treaties were signed during the 1990s and the first decade of the 21^st^ century to introduce cooperation in other areas, and new nations joined the EU. By 2016 EU Members States numbered 28 countries, including the UK, with Iceland, Liechtenstein and Norway forming part of the single market termed the European Economic Area (EEA), but not full members [18].

Today the EU is a unique economic and political partnership between its member states that cooperate on trade, security, defense, foreign policy and legislation [19]. Its existence has marked a period of peace across the EU. However, its growth has not been without challenges. For example, the global financial crisis triggered by the collapse of Lehman Brothers, contributed to economic problems in the EU (the ‘Eurozone Crisis’) [20]. Responses to this crisis were problematic for those countries tied into the Euro with Greece receiving a number of ‘bail out loans’. In addition, since 2014 the EU has faced a crisis in the numbers of refugees and migrants travelling to the region to seek asylum [21].

### The Brexit referendum

The UK made its first application to join the EEC in 1961 under the Conservative Prime Minister Harold Macmillan, and its second application under Labour Prime Minister Harold Wilson in 1967. Both applications were blocked by the French President, Charles de Gaulle. Its third application in 1973, under Conservative Prime Minister Edward Heath, was successful. On 5 June 1975 a referendum was held to confirm the UK’s continuing membership of the EEC. 64% of citizens eligible to vote turned out. The British citizens voted convincingly to stay in (67% to 33% of those who voted).

A number of EU referenda have been held in Member States, related to joining the EU and ratifying particular treaties intended to extend its powers and their nature [18]. Whilst Greenland, a territory of Denmark, voted to leave the EU in 1982 (52/48 in favor of leaving based on voter turnout), finally separating in 1985, the UK is the first Nation State to hold a vote to leave.

The promise of an EU referendum was made in the Conservative Party’s Manifesto for the 2015 UK General Election [22] under the leadership of David Cameron, Prime Minister at the time. It was made in the context of controlling immigration and reforming the way the EU operated, which was described in the Manifesto as “too big, too bossy and too bureaucratic” [22] (p.72). The aim was to “commit to keeping the pound and staying out of the Eurozone, reclaim power from Brussels… and safeguard British interests in the Single Market” and “back businesses to create jobs in Britain by completing ambitious trade deals and reducing red tape” [22] (p.72). It was to effect “real change” in the UK’s relationship with the EU.

On the 23^rd^ June 2016, one year earlier than set out in the 2015 Manifesto, the citizens of the UK (England, Northern Ireland, Scotland and Wales) and Gibraltar were given the opportunity to vote ‘leave’ or ‘remain’ to the single question ‘Should the United Kingdom remain a member of the European Union or leave the European Union?’ British, Irish and Commonwealth citizens resident in the UK or Gibraltar and aged 18 years old on the 23^rd^ June 2016 were eligible to vote. UK citizens resident overseas were also eligible to vote provided they had been registered to vote at a UK address in the previous 15 years.

LeDuc [23] notes that referenda are, in theory, a democratic process which is not party political, but nevertheless factions and parties often do play a significant role. In the UK campaign, Parliamentary party members were given a free choice to lobby in line with their personal beliefs. Multiple ‘leave’ and ‘stay’ campaign groups formed with differing agendas and promises. This complicated voter choices as it was unclear what ‘leave’ meant. Key issues were whether the vote would result in the UK leaving the EU legislative process, the EEA and free trade agreements, and the free labor market/movement [24]. In these contexts, Gibraltar had particular concerns regarding the continuation of trade and travel across the continent, whilst Northern Ireland did not want a ‘hard’ border with the Republic of Ireland. The detail of what leaving the EU meant remained unresolved at the point of the research data collection. Nevertheless, it is clear the UK decision to ‘Brexit’ represents a significant change for the UK, and has further reaching impacts beyond the UK.

In an unusual step, global leaders and business leaders throughout the world commented ahead of the vote with some urging UK voters to vote to stay in the EU. For example, during a visit to the UK in May 2016 the Japanese Prime Minister Shinzo Abe warned that leaving the EU would be a mistake, which would impact on Japanese businesses such as Hitachi [25], and the US President Barack Obama spoke on the benefits of a ‘remain’ vote during his visit the week before the Referendum [26]. These interventions indicate the connectivity between industrial and national economics and the desire for stability at a transnational political level.

**Fig 1:**
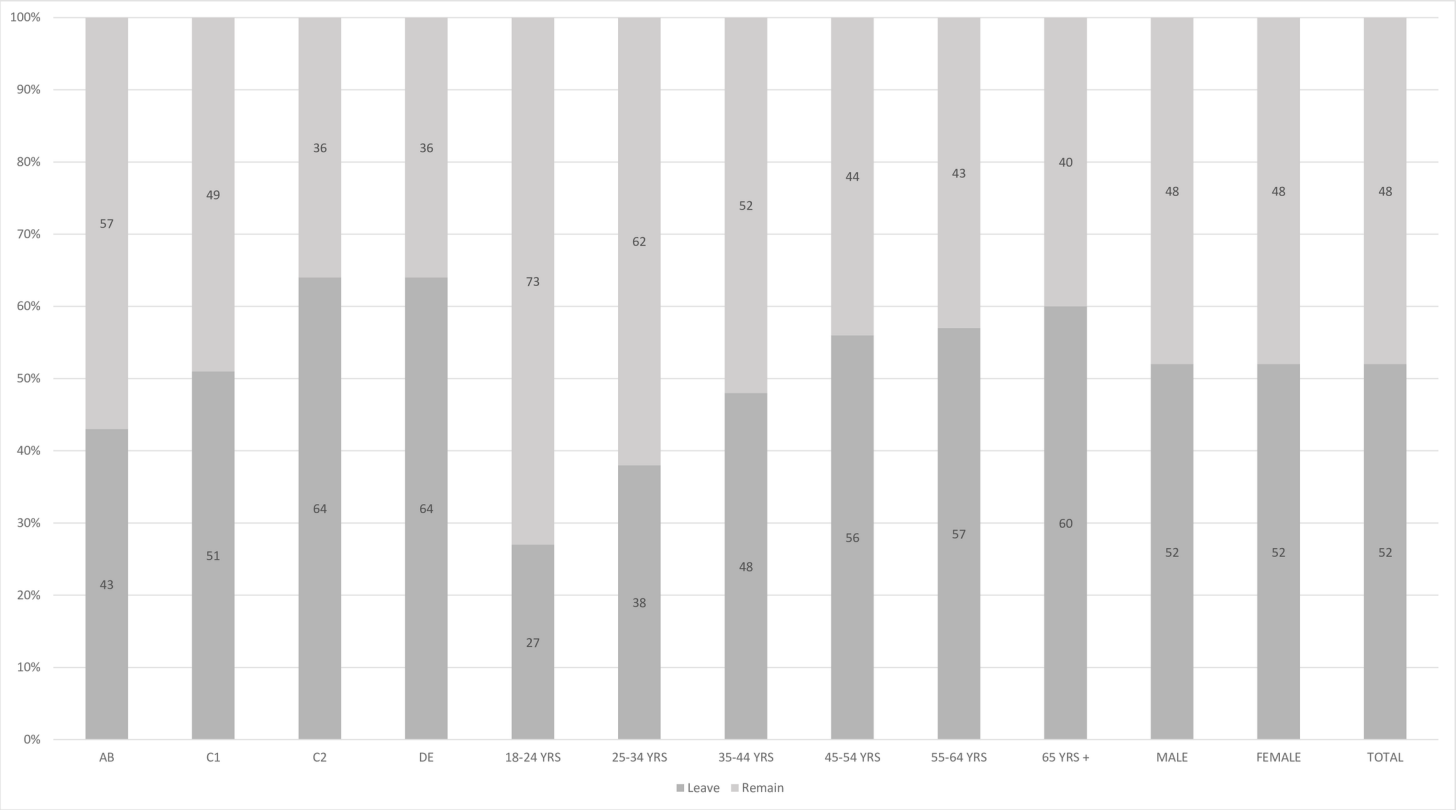
Representation of data from Lord Ashcroft’s poll breaking down demographics on the Brexit vote [29]. Key: AB, C1, C2 and DE are social grades defined by the National Readership Survey (www.nrs.co.uk/nrs-print/lifestyle-and-classification-data/social-grade/Survey) ranging from AB (upper middle and middle class) to DE (semi/unskilled, state pensioners and others).

Following a voter turnout of 72%, on the morning of Friday 24^th^ June 2016 a victory for the ‘leave’ camp was declared with 51.9% of the votes across the UK being cast in favor of leaving the EU [27]. Regionally the voter breakdown indicated that England and Wales were in favor of leaving the EU with Gibraltar, Scotland and Northern Ireland in favor of remaining within the EU. However, the Referendum outcome was determined purely on adding the total number of votes from across the UK rather than any regional considerations. As the process was a secret ballot, a more detailed understanding of the breakdown of voter demographics is reliant upon polls, which indicated that age, managerial status and educational background influenced voter choices (Fig 1). *The Guardian* [28] mapped the voting percentages against regions where a higher percentage of the residents had a higher education and found that these areas were more likely to have voted ‘remain’.

## Methods

### Survey and analytical framework

The data was collected via a survey (S1 Fig) developed and administered using the online survey tool SurveyMonkey (https://www.surveymonkey.net/). The survey was open to anyone across the world since, although Brexit was a UK vote, it was important to understand how those outside the UK understood the threats and opportunities for ICT professionals which might arise in the light of Brexit. Whilst Brexit itself was an emotive issue, contributions were requested to be made in a constructive manner even when noting concerns.

In order to aid the protection of the identities of those who participated, IP address data was not captured. Furthermore, the number of personal data fields was limited to attempt to minimize the potential for individual identification; only the participant’s place of residence and nationality were collected for demographic purposes, to place the responses in context. The survey provided an opening consent page which explained its purpose. All 20 questions were optional. In order to enable free and frank contributions permission was not sought for the full data set to be shared. All the qualitative data quoted was carefully manually reviewed in order to protect both individuals’ identities and organizations’ commercial confidentiality as well as taking into consideration issues around sensitive personal data such as political opinions.

The survey was purposefully designed with a mix of quantitative and qualitative questions. A STEEPLE model was used as the central data collection framework, specifically focusing on opportunities and threats. STEEPLE is an acronym for seven factors—Socio-cultural, Technological, Economic, Environmental, Political, Legal and Ethical. This model is a common management tool used in commercial circles [30], including ICT settings [31]. It provides a basis for strategic planning and risk review and is seen by some as being more advanced than similar analytic tools “as it deals with macro-environmental external factors” and “offers an overview of various external fields” [32]. STEEPLE analysis allows businesses “to anticipate future trends by considering the macro environment in which a company operates, enabling it to determine the factors that will influence it in the coming years” ([33] p.2). It has also been “frequently compared with and paired with the Strengths, Weaknesses, Opportunities and Threats (SWOT) analysis”([34] p.2). In this research, the STEEPLE descriptors were adapted from Harcourt’s model [35]. Dr Oliver Duke-Williams, UCL, is to be acknowledged for providing advice on the questionnaire design.

The survey was piloted on five researchers and five practitioners from the target audience before being formally launched on Monday 27^th^ June 2016, the first full working day after the UK vote to leave the EU had been announced. It was distributed globally by the authors using a virtual snowballing (i.e. referral) sampling method [36] via direct email, email listservs, online newsletters and social media posts (e.g. Facebook, LinkedIn and Twitter). The survey remained open for exactly four weeks closing on Monday 25^th^ July. Reminders were sent out in the final week before it closed. During the period of data collection, research participants could amend or add to their answers. The data was analyzed using the tools available in SurveyMonkey together with Excel and AtlasTI (http://atlasti.com/). An inductive process to coding the qualitative responses in AtlasTI was adopted, utilizing the steps set out by Lewins and Silver [37].

The research was a collaboration between University College London and Northumbria University and was given ethical approval by both universities.

### Survey demographics

A total of 733 participants completed the survey. 520 of the responses were received in the first week of it being open. The authors received a number of telephone calls and emails in response to the survey. These relayed how upset people were following the decision, sympathy to the authors from non-UK residents/nationals following the decision, and queries relating to whether the UK job market would now offer greater opportunities to non-EU nationals. In addition, a number of people noted that they did not consider this an appropriate subject for research at that time and perceived it as an inappropriate focus for a survey.

**Fig 2:**
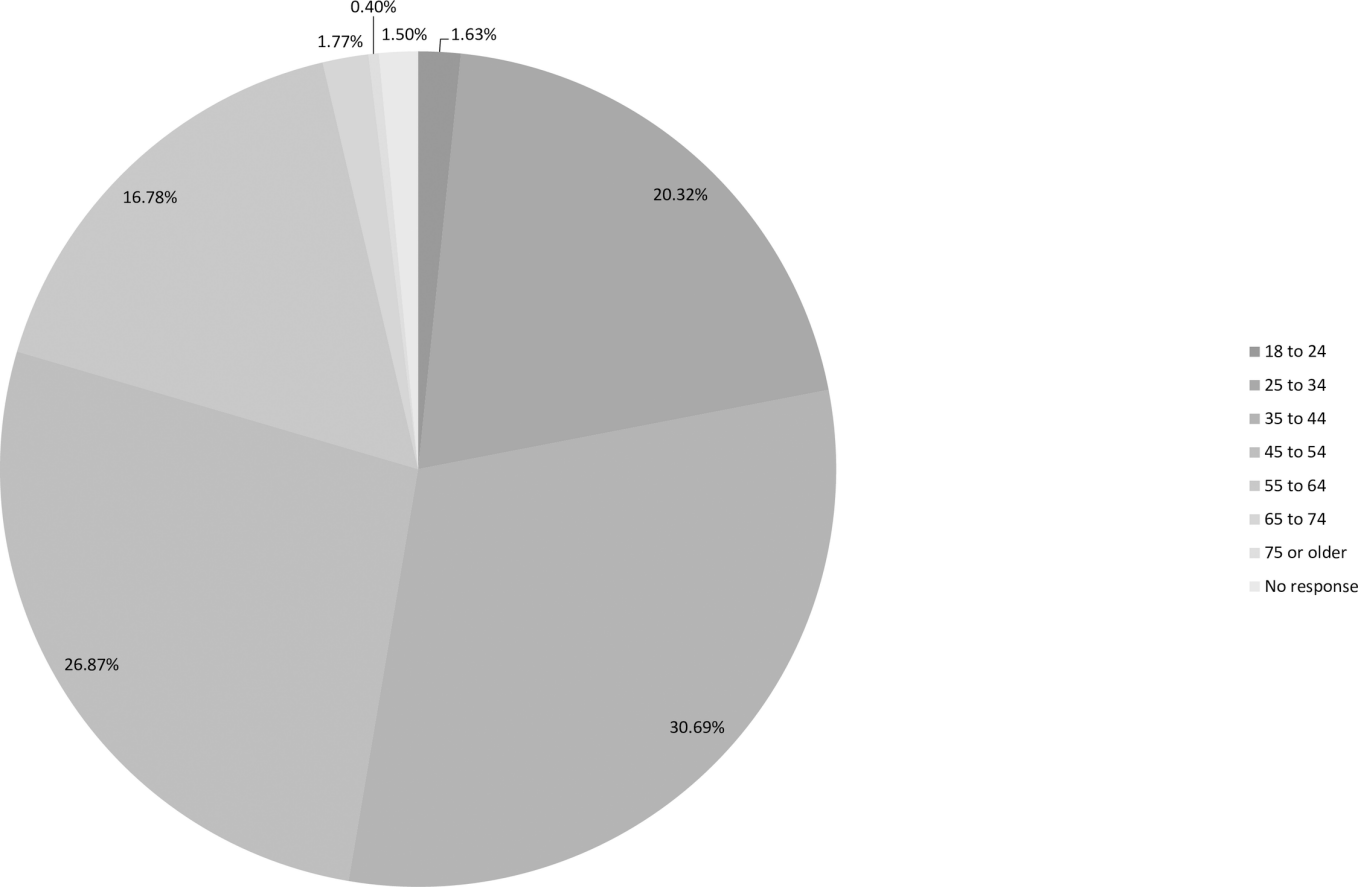
Age distribution of participants (S1 Table).

**Fig 3:**
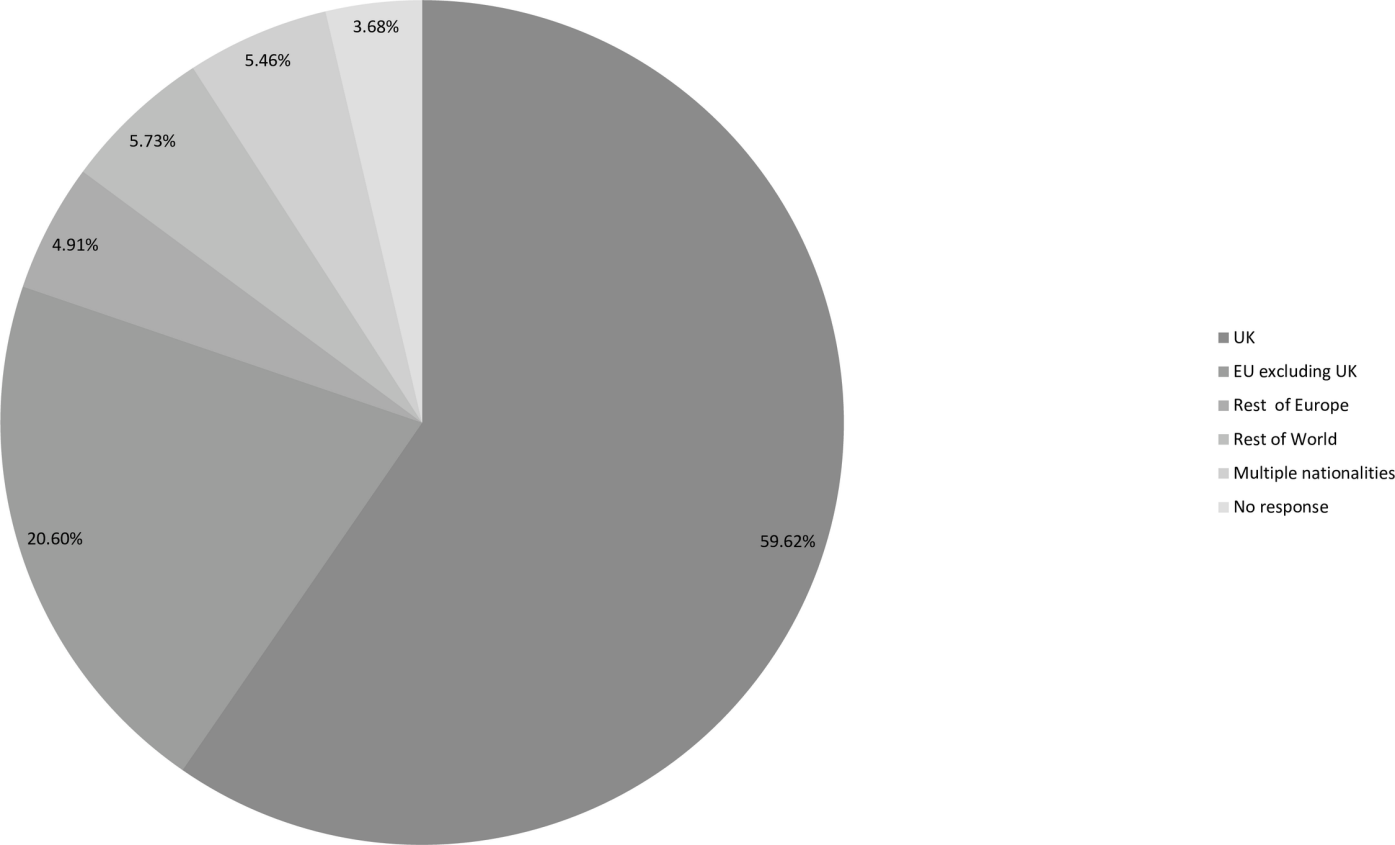
Nationality profile of participants (S2 Table).

**Fig 4:**
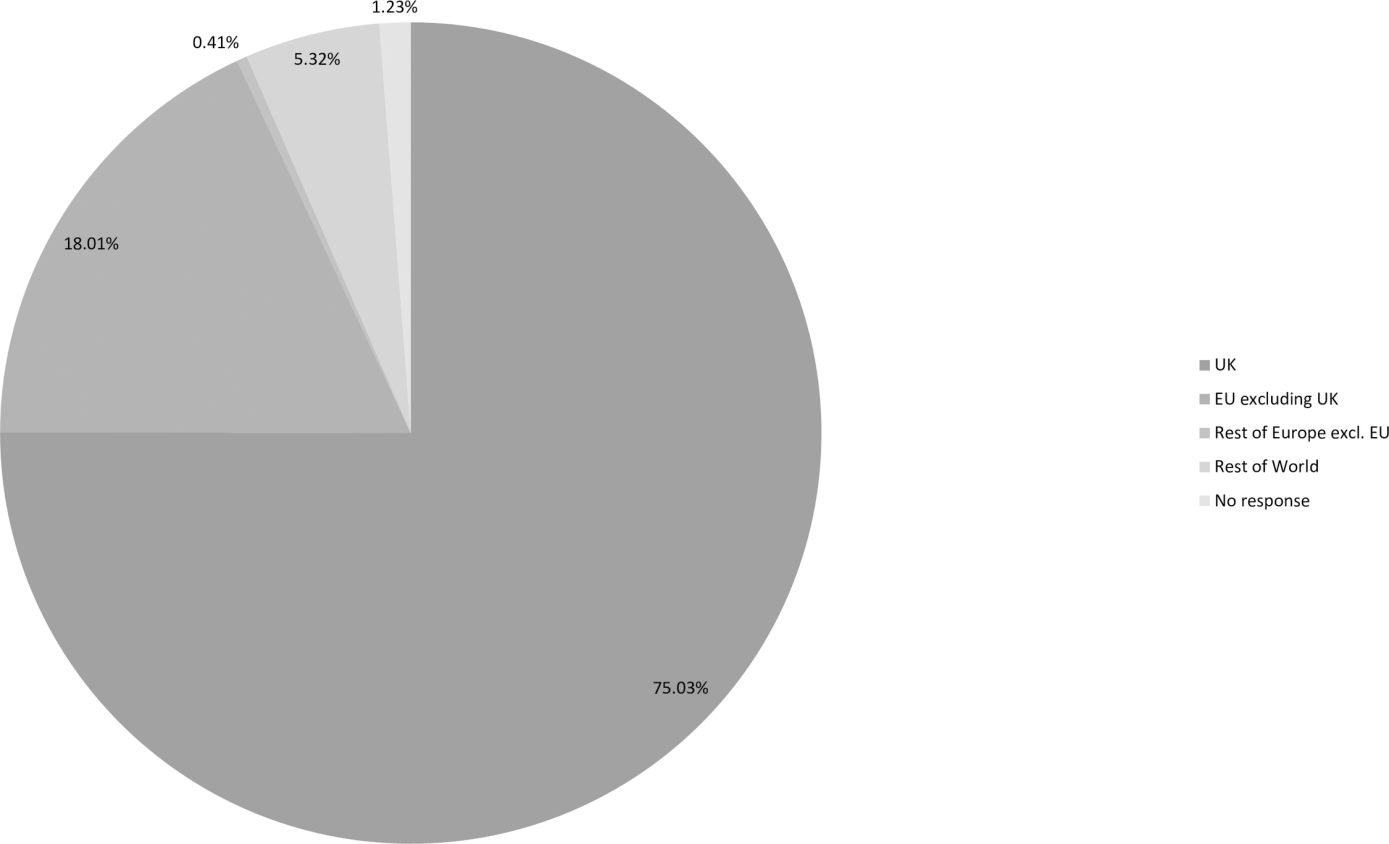
Residence profile of participants (S3 Table).

Figs 2, 3 and 4 (S1, S2 and S3 Tables) indicate the age, nationalities and locations of those who responded. Despite being circulated globally the majority (75%) of participants were based in the UK and the responses to a separate question showed that they were spread across all parts of the UK. 59% of participants were UK nationals with 2% resident outside of the UK. Only four people responded from Belgium, although it is considered the *de facto* headquarters of the EU, two of whom were UK nationals. The highest number of responses, excluding UK nationals, was from German nationals who accounted for 7%, followed by the Republic of Ireland nationals (4%). Excluding EU nationals, the highest number of responses was from Australians (2%), followed by the USA (1.5%). Thus, despite the reach of this issue the highest level of engagement was with UK based participants. This may in part be due to the timing being the day immediately after the referendum decision and also because the survey was only distributed in English, which will have influenced its reach in terms of the nationalities and education of those who responded.

Table 1 indicates the professional status of the participants and their employment contexts. 48% were managers, 28% were working in global enterprises, and many others noted in the comments fields that their organizations, whilst located in one country, managed information and markets across boundaries. 65% worked in the public sector with 44% of these being from within the higher and further education (HEFE) sector, which provides education for those over 16 years of age. The HEFE demographic may have influenced the responses in terms of the coding counts for issues related to students, education and research. In addition, a large percentage of librarians (24%), working in a rage of contexts across the public and private sectors, responded.

**Table 1:** Professional status and employment contexts of participants.

Professional status for participants	%	Employment contexts for participants	%
**Occupational status**		**Number of employees in organization where participants were employed/ volunteered**	
Employee	86%	0–9	7%
Self-employed	5%	10–49	8%
Unemployed	1%	50–249	14%
Student	5%	250–999	20%
Volunteer	1%	1000–9999	41%
Retired	1%	10000+	10%
Other	1%		
**Management role**		**Organizational sector**	
Management role	48%	Public Sector	65%
No management role	51%	Private Sector	30%
N/A	1%	N/A	5%
**Profession/ job function/ area of study**		**Global organization operating from sites across the world**	
Administrator	2%	Yes	28%
Archivist	15%	No	72%
Business analyst	1%		
Cyber security/ information security	3%		
Data manager	3%		
Information manager	18%		
IT support	3%		
IT Project manager	12%		
Lawyer	1%		
Librarian	24%		
Management consultant	1%		
Marketing manager	1%		
Mergers and Acquisitions expert	0.5%		
Software developer	2%		
Records manager	10%		
Telecoms manager	0.5%		
Web designer	1%		
Other	2%		

## Results and discussion

### Plans post Brexit

In terms of organizational contexts, 53% of participants indicated that there were no known plans in place relating to a response or need for action in light of the Brexit decision and that developments were at that stage being monitored. In the words of one participant they were ‘sitting tight’. 26.5% did not know what their organizational response was at the point of the survey or if there would be one, given that in some instances the participants were outside the UK. 6.5% of participants confirmed that their organization had made proactive plans ahead of the Brexit decision in order to deal with a range of eventualities. From within and outside the UK these included exploiting currency fluctuation opportunities and the potential to foster new trading links. Across UK based organizations, 14% indicated that in the immediate aftermath steps were being taken to map out the issues. These included profiling staff in case of changes in terms of employment laws and mapping current and future job market issues/potential. 3.5% of these UK based participants indicated that notices had been sent to all staff and wider stakeholders confirming their organization’s pro-EU and international outlook. These messages were largely aimed at reassuring the non-UK nationals about their value within the organization. 2% of the 3.5% were from the HEFE responses where it was seen that this issue would impact significantly on current staff and students. A small number of participants (5) noted that their organizations were considering relocation to Ireland, France, Portugal, Romania or potentially Scotland.

### Perspectives on the Brexit referendum outcome

**Fig 5:**
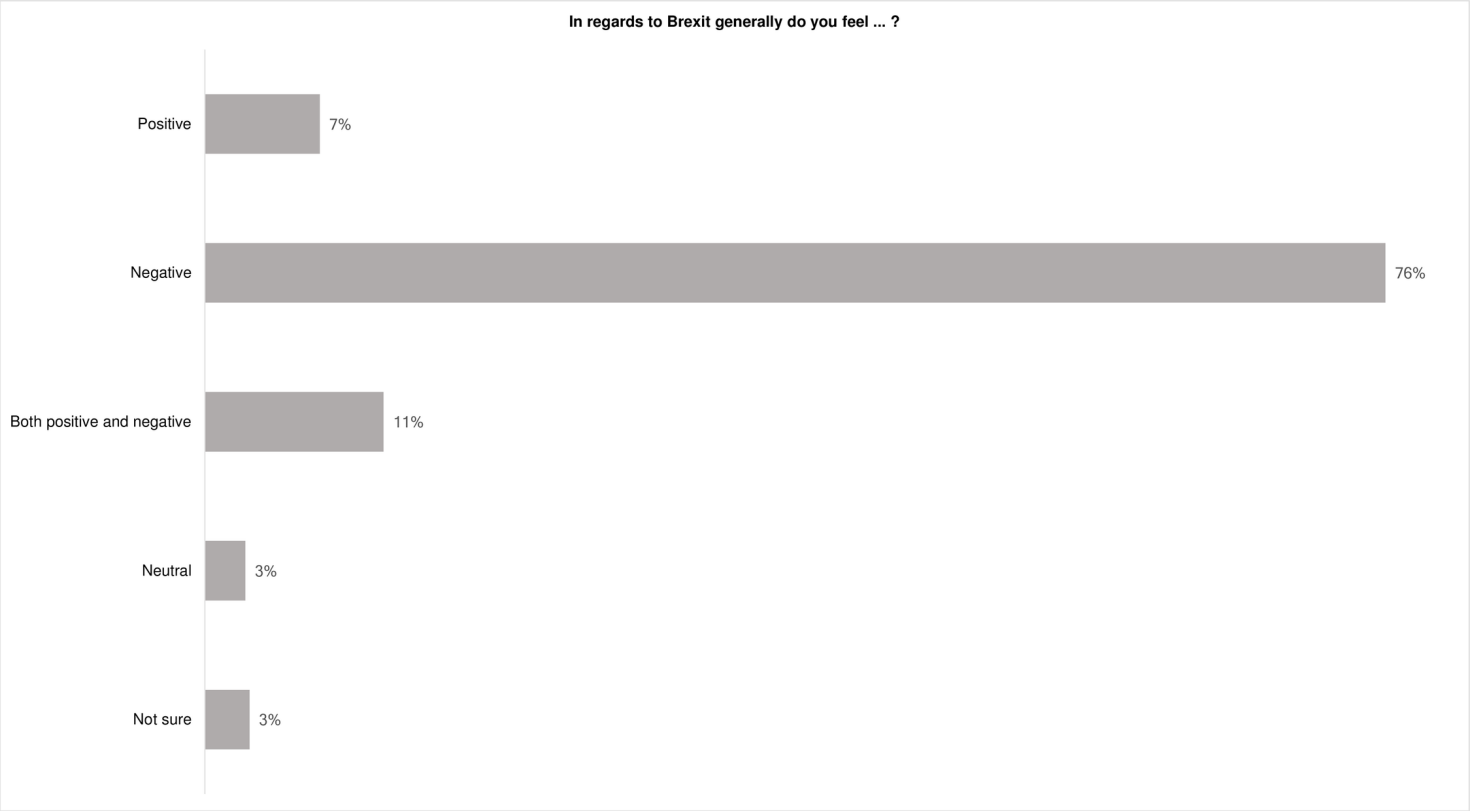
Personal perspectives on the Brexit decision generally.

**Fig 6:**
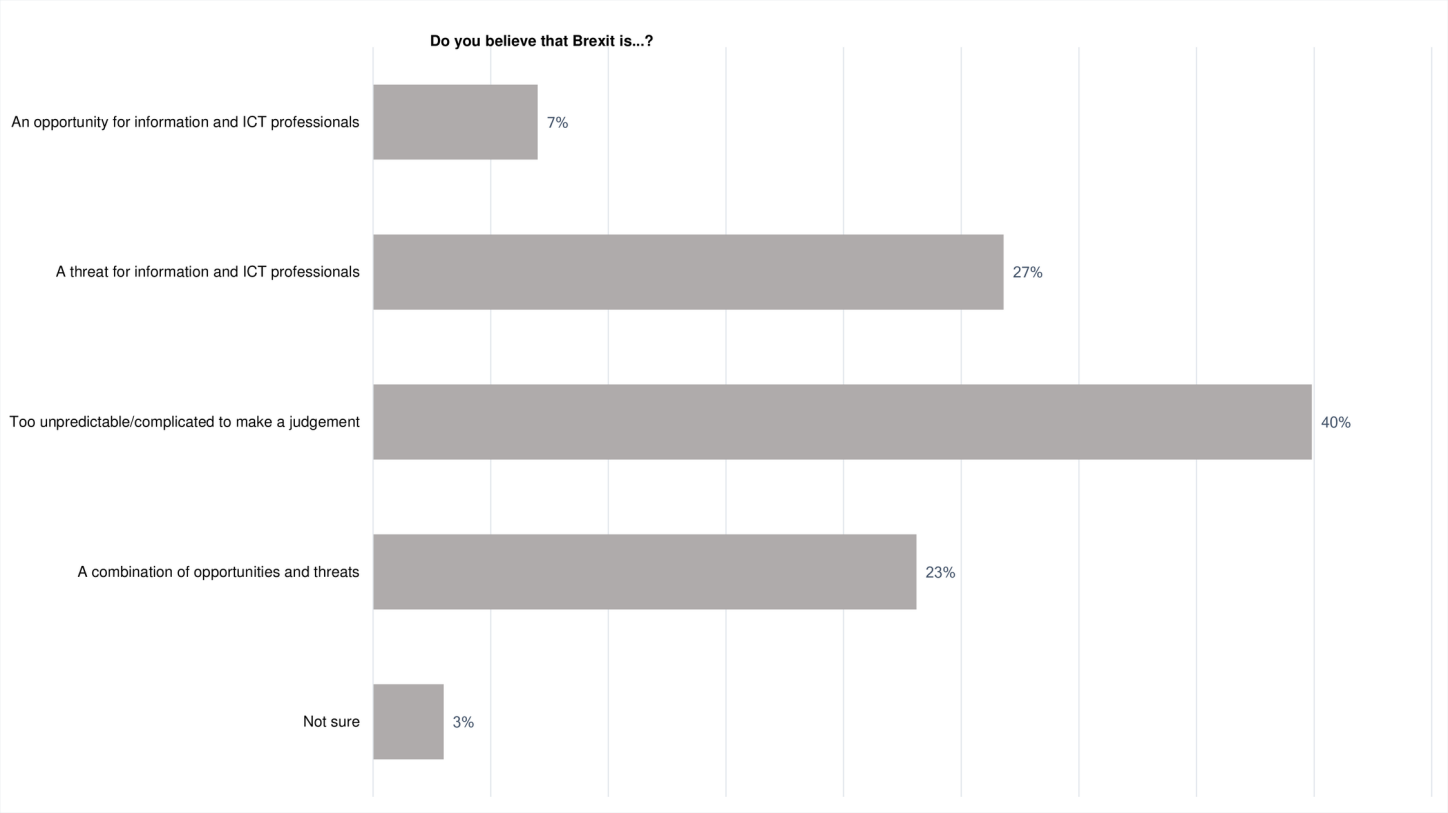
Perspectives on the Brexit decision for ICT professionals.

76% of participants personally viewed the Brexit decision negatively (Fig 5). The ICT community is traditionally banded in AB social groups (upper middle and middle class, professionals and managers) and many ICT professionals have undertaken higher education. These statistics therefore align to the pollster data which indicated that those areas where the residents were more likely to have undertaken higher education were more likely to vote remain [28]. However, Fig 6 indicates that whilst many participants were negatively disposed towards the vote to leave the EU many understood that its likely impacts would be too unpredictable or complicated to make a judgment on the impact of the decision for ICT professionals.

A high number of participants (49%) did not identify any opportunities or threats against the seven STEEPLE factors, with many reporting that it was too early to comment or that more research needed to be done. 13 participants consistently reported negatively regarding the leave agenda in all the comment and question fields. The remaining comments, regardless of their personal outlook and overall sense of the impact of this decision, did identify a mixture of opportunities and threats. In fact, whether the participant was personally positive or negative to the overall Brexit decision did not impact on his/her ability to subsequently review and identify opportunities and threats for ICT professionals.

### STEEPLE summary

**Fig 7:**
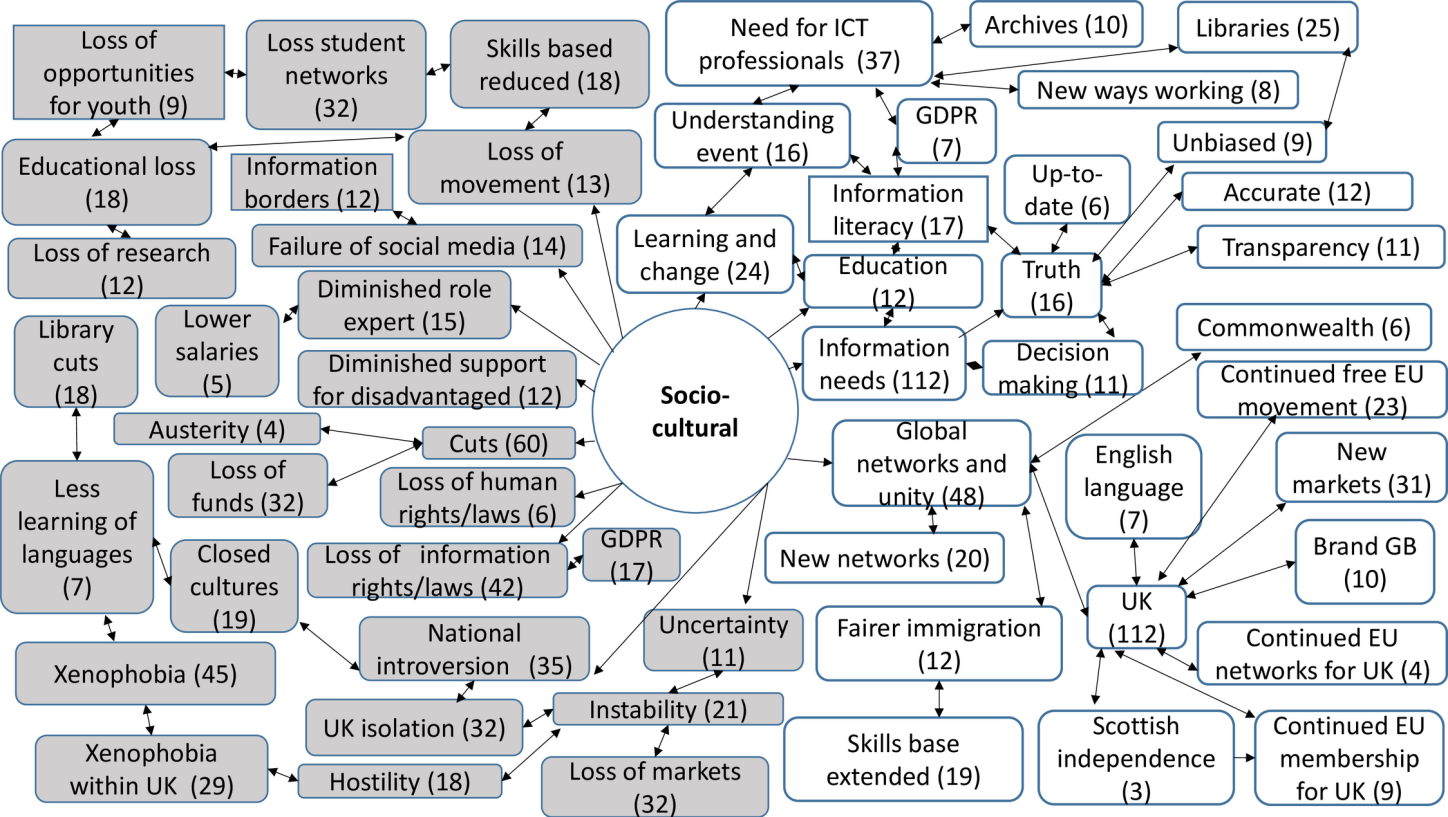
Socio-cultural opportunities and threats: Frequency of code counts. White boxes are coded opportunities and grey boxes represent threats.

**Fig 8:**
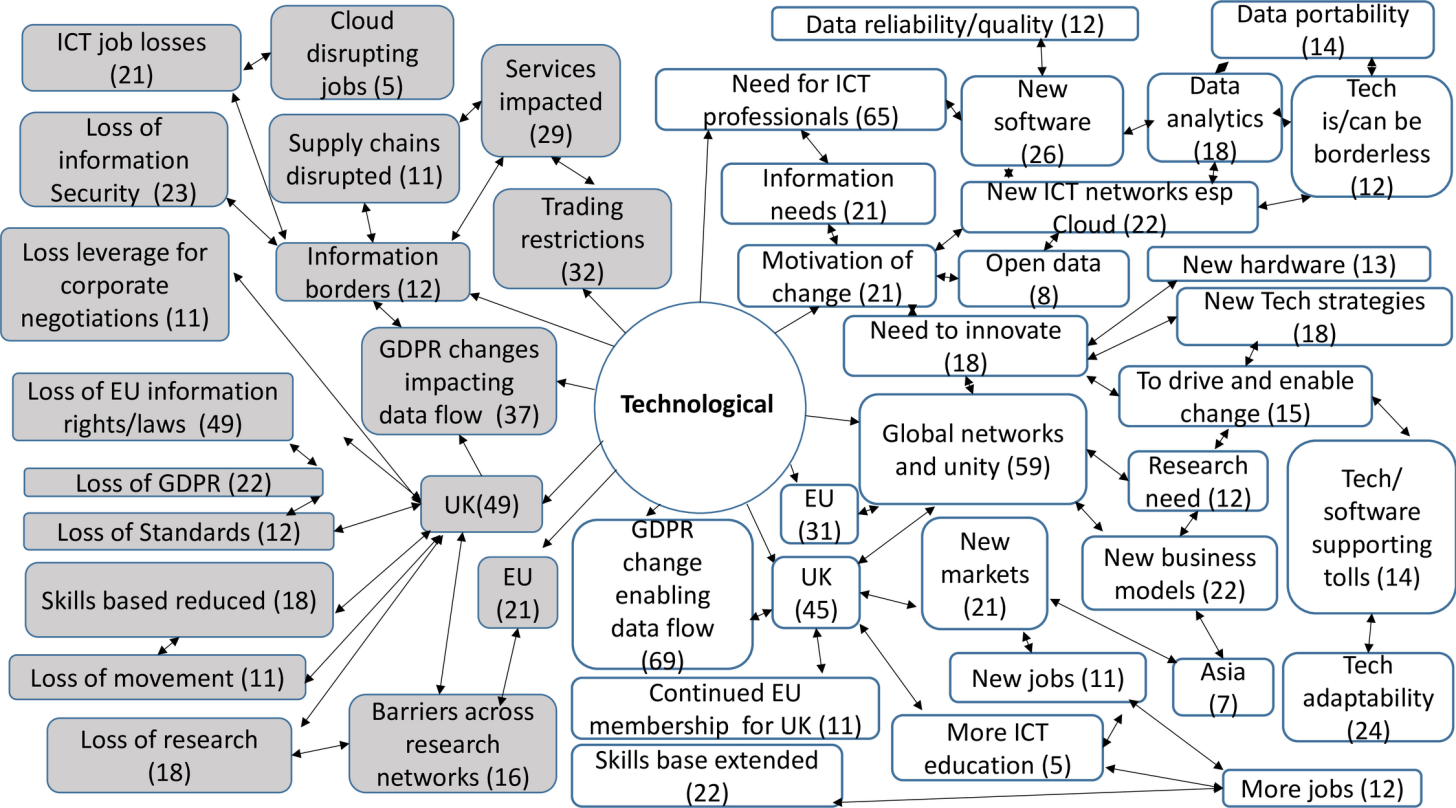
Technological opportunities and threats: Frequency of code counts. White boxes are coded opportunities and grey boxes represent threats.

**Fig 9:**
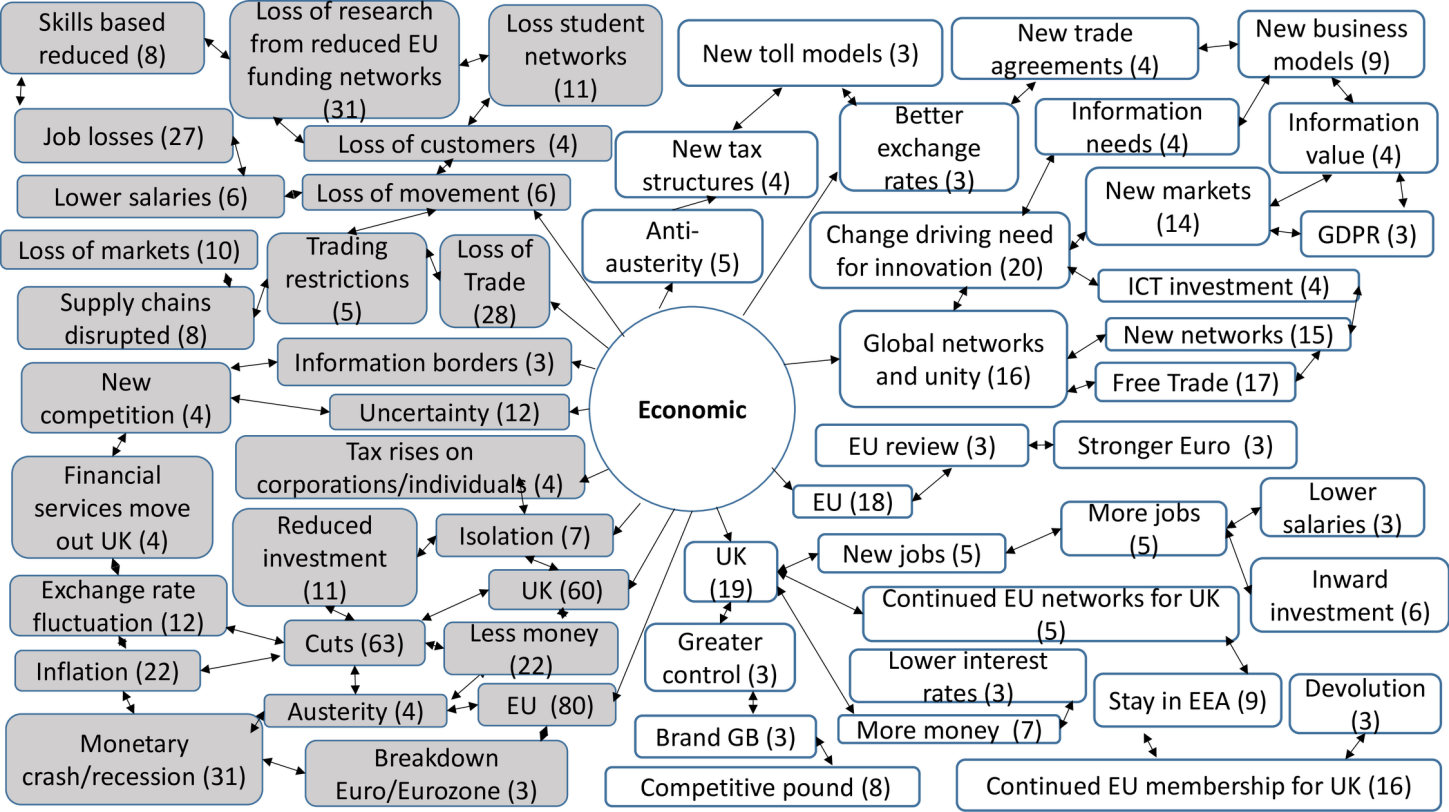
Economic opportunities and threats: Frequency of code counts. White boxes are coded opportunities and grey boxes represent threats.

**Fig 10:**
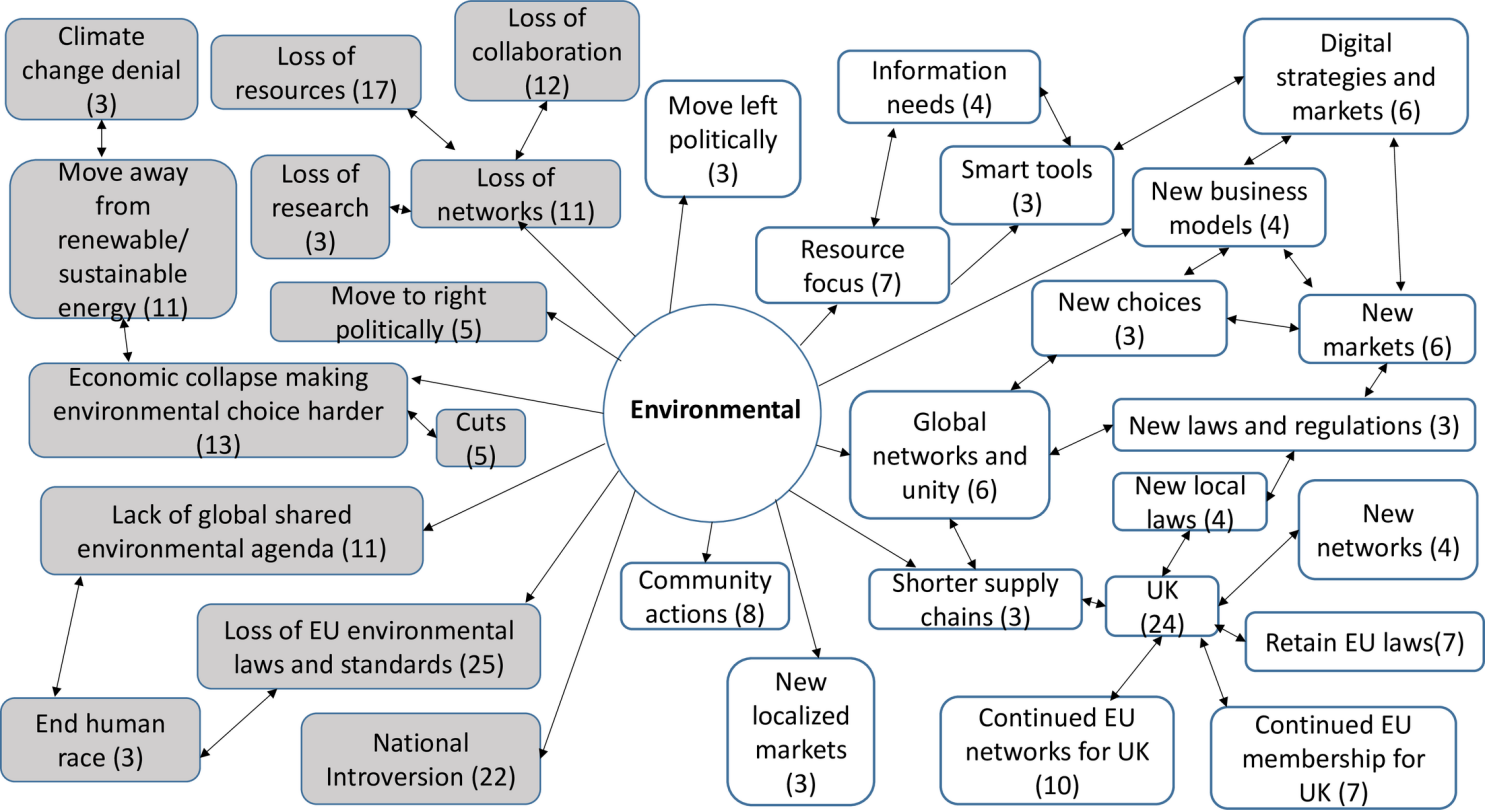
Environmental opportunities and threats: Frequency of code counts. White boxes are coded opportunities and grey boxes represent threats.

**Fig 11:**
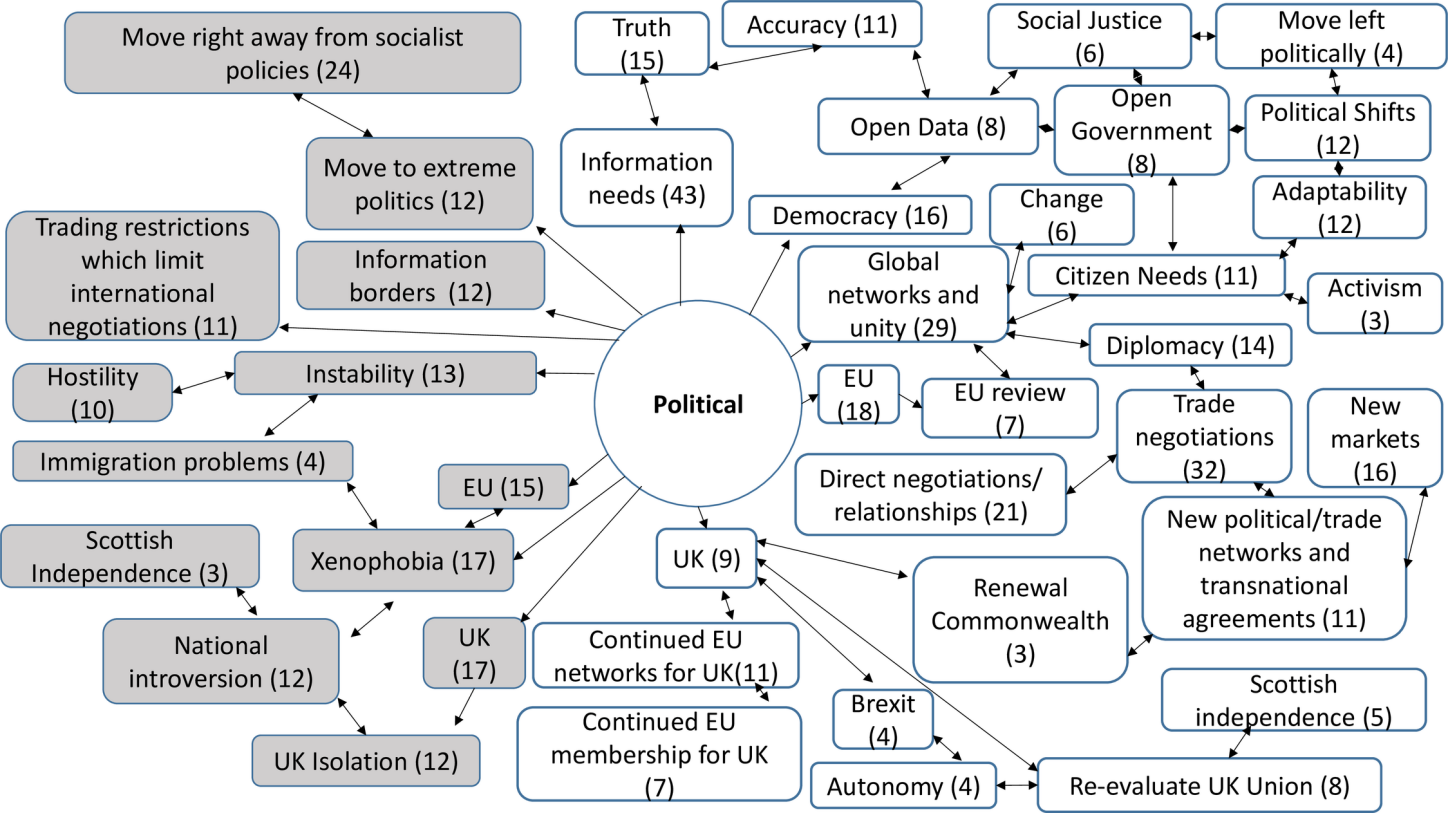
Political opportunities and threats: Frequency of code counts. White boxes are coded opportunities and grey boxes represent threats.

**Fig 12:**
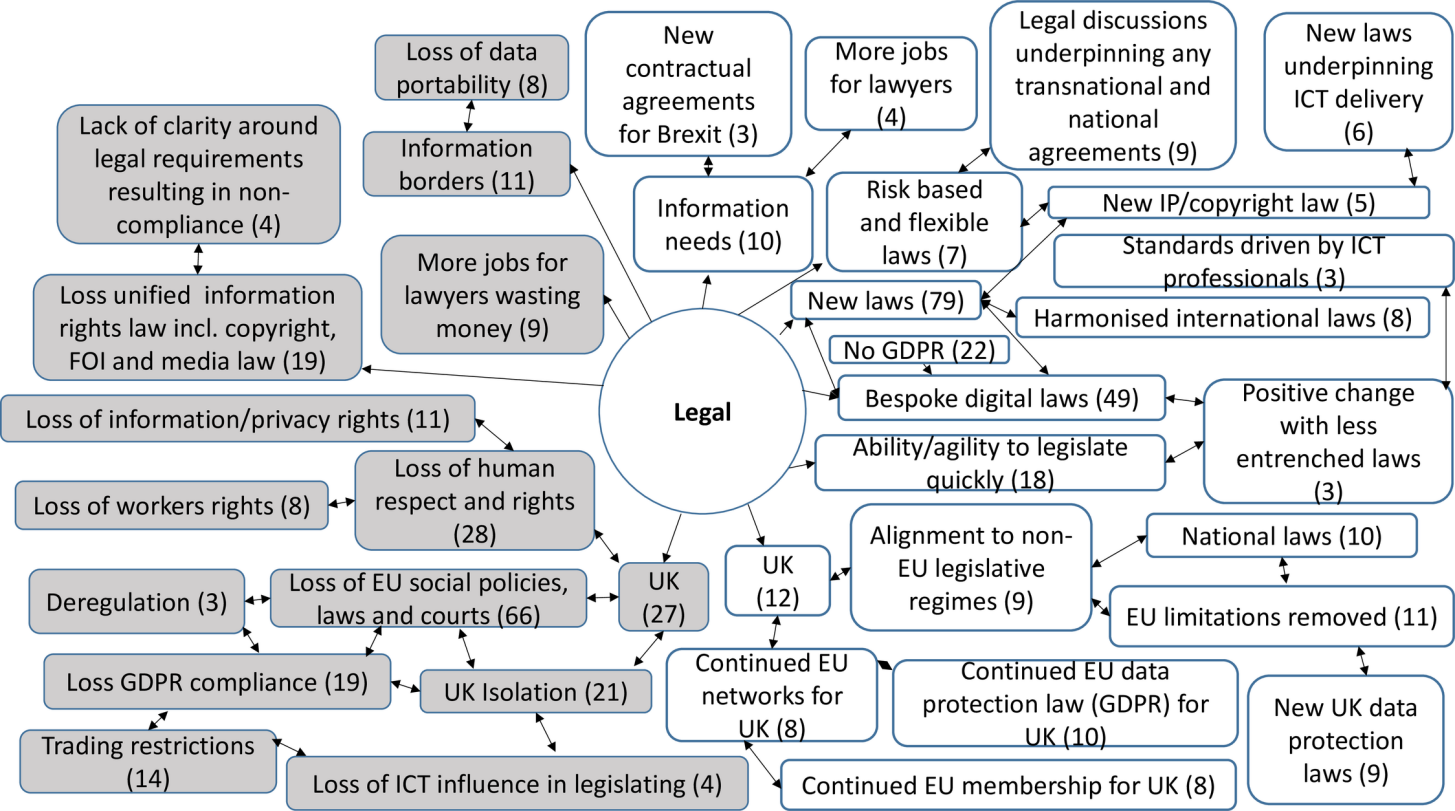
Legal opportunities and threats: Frequency of code counts. White boxes are coded opportunities and grey boxes represent threats.

357 codes were created from the analysis of the responses to the open questions. 30 codes were linked to both opportunities and threats, 219 codes were linked only to opportunities, whilst 108 were linked only to threats. The comments revealed a rich picture of threats and opportunities for the ICT sector and ICT professionals (S4 Table). They also provided an insight into wider attitudes to the decisions and implications for change, which were prompted by the contextual definitions of each factor. Across the STEEPLE model many responses noted the connections and links between the issues that arose under different factors. This meant that sometimes comments were repeated, or connections were made back to earlier narratives. The highest numbers of responses were provided under the first two factors presented—social-cultural and technological. Figs 7, 8, 9, 10, 11 and 12 indicate those codes created against each factor where the code count was three or higher. The codes on the right-hand side relate to the perceived positive opportunities which had been coded against a factor, and the grey colored codes on the left relate to the perceived threats. The arrows provide a visualization of the connections between codes as identified within the responses. Given that Brexit was a UK based decision, many of the coded opportunities and threats related to the UK change context. The UK context is highlighted when relevant.

#### Socio-cultural analysis

Socio-cultural factors (S) include customs, lifestyles, and values that characterize the society in which an organization/individual is operating. They can also include demographics of age distribution, population growth rates, level of education, distribution of wealth and social classes and living conditions. Socio-cultural factors may influence entrepreneurial spirit, fashions and consumer demands, the ability of a society to obtain resources or trade in certain ways due to consumer influence (Fig 7).

#### Technological analysis

Technological factors (T) refer to the rate of new inventions, development and changes in technology including software, hardware, networks and e-commerce. They can also include: methods of manufacture, distribution and logistics. These factors also include attitudes to research and research spending (Fig 8).

#### Economic analysis

Economic factors (E) represent the wider economy so may include: sector growth, levels of employment, consumer confidence, costs to the sector (e.g. hardware and licenses), interest rates and monetary policies, exchange rates, inflation, investment opportunities and research funding (Fig 9).

#### Environmental analysis

Environmental factors (E) include energy and resource-efficient goods (carbon neutral, recycling etc.) services and technologies and promotion of informed choices by customers. They also include threats from natural disasters (Fig 10)

#### Political analysis

Political factors (P) refer to the stability of the political environment and the approaches of political parties, sector interest groups and other stakeholders. This may include policies on tax, trade or the sector in general (Fig 11).

#### Legal analysis

Legal factors (L) include for example, domestic legislation, EU regulation, self-regulation, standard agreements, international trade agreements, national and international competition law, trade union agreements and consumer protection (Fig 12).

#### Ethical analysis

**Fig 13:**
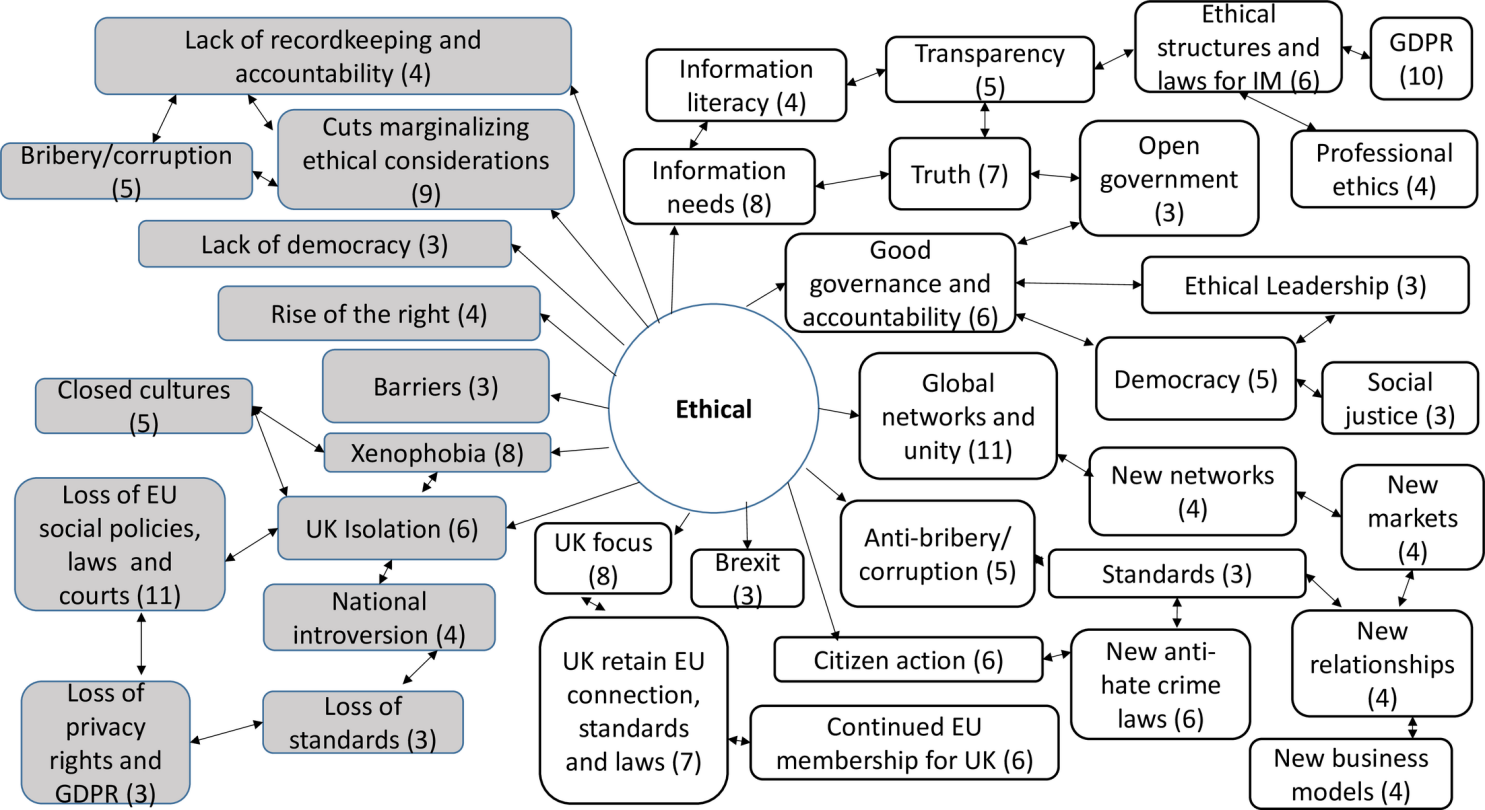
Ethical opportunities and threats: Frequency of code counts. White boxes are coded opportunities and grey boxes represent threats.

Ethical factors (E) include the moral expectations determining how individuals and organizations should operate. This is the domain of governance which extends beyond the strictures of law and influences approaches to accountability and confidentiality, bribery, intellectual property rights, brand and reputation (Fig 13).

## Emergent issues

### Opportunities and threats

The data revealed a rich and complex picture. Whilst for a majority of participants the outlook was bleak in the immediate aftermath of coming to terms with this decision, many noted the complexity of the decision and saw both opportunities and threats. The coding frequency ratio for opportunities compared to threats was 1:2.02. A number of emergent themes cut across more than one of the STEEPLE factors. Table 2 shows the top 10 themes, in terms of coding frequency, for both opportunities and threats. It interrelates to Figs 7, 8, 9, 10, 11, 12 and 13 which indicate connections between the themes including sub-themes. The majority of the top threats identified relate to wide ranging societal impacts. The opportunities identified focus much more specifically on the ICT context and areas where ICT professionals could contribute and control events. Drawing on the coded themes, this wider context is discussed before looking more explicitly at the ICT context. Coding frequencies from Figs 7, 8, 9, 10, 11, 12 and 13 and Table 2 are included in brackets within the discussion.

**Table 2:** Highest coding counts for the codes that cut across more than one factor.

	**Opportunities**	**S**	**T**	**E**	**E**	**P**	**L**	**E**	**Total**
1	Information needs	112	21	4	4	43	10	8	202
2	Global networks and unity	48	59	16	6	29	0	11	169
3	Need for ICT professionals	37	65	0	0	0	0	0	102
4	GDPR delivered widely incl. UK	7	69	3	0	0	10	10	99
5	New markets	31	21	14	6	16	0	4	92
6	GDPR altered/not implemented	0	69	0	0	0	22	0	91
7	New laws excl. GDPR	0	0	0	4	0	79	0	83
8	Continued EU membership for UK	9	11	16	7	7	8	6	64
9	New networks	20	0	15	4	0	0	4	43
= 10	Continued EU networks for UK	4	0	5	10	11	8	7	41
= 10	Skills based extended	19	22	0	0	0	0	0	41
	**Threats**	**S**	**T**	**E**	**E**	**P**	**L**	**E**	**Total**
1	Loss of EU social policies, laws and courts	48	49	0	25	0	66	14	202
2	Cuts	78	0	63	5	0	0	9	155
3	Loss of information rights incl. GDPR	42	49	0	0	0	38	3	132
4	Isolation	32	0	7	0	12	21	6	78
5	National introversion	35	0	0	22	12	0	4	73
6	Xenophobia	45	0	0	0	17	0	8	70
7	Loss of research	12	18	31	3	0	0	0	64
8	Trading restrictions	0	32	5	0	11	14	0	62
= 9	Information borders	12	12	3	0	12	11	0	50
= 9	Job losses	2	21	27	0	0	0	0	50

#### UK relationship with the EU

Not all the participants were certain that the Brexit result would lead to a UK departure from the EU. 64 comments related to the opportunity for the UK to continue its membership of the EU, including:

Stay in the EU (?)

Avoid Brexit, stay in the EU.

There will be a recession, so less resources for ICT, and there is nothing we can do to prevent it unless we are prepared to take on the significant political challenge of overturning the referendum decision.

Other comments included a hope that the UK would stay in the EEA (9) or at least remain strongly networked and connected to the EU trading and political regimes (41). A high number of the participants (202) noted that the loss of EU membership could result in the loss of EU social policies, laws and justice systems. This was seen to raise the risk of losses of human rights including access to public sector information and privacy rights (132). Aligned to these values, a further threat of Brexit was perceived to be if it moved the UK away from the EU’s ideologies towards radical right wing politics, such as those deemed to be represented by Donald Trump (12). However, there were participant comments strongly in favor of Brexit (12). Participants noted that the Brexit change could pave the way for more left wing political ideologies which were flagged as opportunities (4). In particular, it was noted by five participants that there was an opportunity to promote anti-austerity measures. Fears of more right wing politics were related to cuts in a range of services. There were concerns that this vote would lead to ‘uncertainty’ (23), ‘inflation’ (22), ‘monetary crashes and recession’ (31), ‘job losses’ (50) and ‘cuts’ (155). Job loss and cuts were raised more frequently by those working in the public sector. Related concerns were that cuts to a range of public services which would impact most heavily on those who were already potentially the most disadvantaged within society, such as the disabled and unemployed.

It is important to note that some UK participants did observe potential benefits from the UK leaving the EU. These comments aligned to messaging from the ‘Leave’ campaigns that money previously sent to the EU would be spent in the UK, although in most there was doubt expressed as to whether this would be a reality:

If Leave campaigners are to be believed, more UK money being available to spend on UK research.

There's even a very, very slight chance that if promises of greater national spending on UK come true then we may be able to invest more in this [ICT] (though I seriously doubt it).

Less money will be spent propping up the EU and supporting foreigners to live in our country. There will be more money and jobs for our own people.

Other perceived opportunities were the chance for the UK to be ‘adaptable’, ‘able to legislate quickly’ and open to ‘new business models’ and ‘new networks’. In this context networks included new kinds of connection between people, countries and businesses.

#### Global networks vs. isolation

Global networks and unity was a key theme that arose throughout the comments with 169 comments coded. These responses highlighted a need to collaborate beyond continents and to have global shared agendas. Whilst the globalized agendas were highlighted much of the commentary then focused on specific regional concerns. From a UK perspective, it was seen as essential for the UK to reach out to a range of partners from Commonwealth nations including Australia, Canada, New Zealand and India, as well as to Asia and ‘the East’ including China, Dubai and Saudi Arabia. In addition, the USA was identified as a key ally despite President Obama’s warnings about the UK’s relationship with the USA if it left the EU. Two Australian participants commented that it had been hugely detrimental to global networks when the UK had decided to enter the EU and that this leave decision was positive in providing the opportunity for the UK to re-establish wider networks. However, one Australian noted that Australia had not forgiven the UK for the ‘1970s betrayal’ when it joined the EU and deserted former trading partners.

The threats of the Brexit decision, in contrast to global working, were perceived to be national introversion (73), closed cultures (24) and xenophobia (70). Concerns about inward facing cultures and xenophobia were not only limited to the UK context. The narrowing of perspectives was also linked to the diminished role of the expert (15).

Anti-intellectualism, xenophobia, growing overseas dislike of UK, diminished environment for R&D

We are seeing everyone narrowing their minds and this is made acceptable by parties like UKIP, the right wing German parties, Trump and others who now think it is acceptable to blame others for any problems and cause conflicts, hostility and war. This must be challenged.

The Brexit vote was seen as closing down some currently occurring cultural exchange. Highlighted were the loss of students (43), research (64) and trade (28) which would all limit the UK’s global networks. Each one was seen to have a linked impact. For example, not only were overseas students a funding source for the country and HEFE sector at the time of the research, but part of the job skills pool and trade network of the future. Likewise, research was identified as creating shared global networks and driving innovation linked to economic growth and trade.

For many people, globalization and connections were seen as driving and providing leadership for ethical behaviors. It was noted, under the environmental and ethical STEEPLE factors, that it can be harder to prioritize and develop ethical standards and systems when resources are scarce (22) and there is limited international cooperation. As such, concerns around the impact of the Brexit decision on the global economy were then seen to have the potential to impact on environmental and wider ethical agendas. In regards to climate change a global response was seen as the only way to address the planetary challenges, with one participant noting that the alternative would be “collapse of natural environment and lights out for the human race.”

In reviewing the status of UK nationality and borders, the data revealed a complex picture with many in favor of immigration but wanting to recognize cultural heritage. A number of continental Europeans based in the UK noted the need to recognize and confirm their rights to stay, work and be supported within the UK. Many made the call for the “guarantee of rights to remain for EU citizens currently in the UK.” The balance of comment was in favor of protecting EU freedom of movement (30). However, an opportunity noted by a number of participants was the fact that the UK could take in a much greater mix of labor and draw from a stronger skills pool (41) if it was more globally connected and introduced fairer globalized immigration systems (12). These comments indicate some of the complexity of surfacing these issues.

Potentially Brexit could bring about a fairer immigration system that allows us to recruit from further afield

Guarantee right to remain for EU citizens currently in the UK; guarantee principle of free movement

In the event that the UK goes to a points based immigration policy there is an opportunity to limit entry to satisfy gaps in the employment market. For example if deficient in a certain skill set / trade, then exceptions can be made to allow these individuals in to the UK from other countries as long as they have these key skills.

There were responses which identified specific opportunities viz. “a UK brand that can be marketed externally” or “brand GB”. However, whilst identity was a concern, global networks were flagged as key for all nations, in order to avoid the worst societal threats including environmental disaster and war:

Expanding networks throughout the country and intentionally, in a truly international way rather than international just meaning Europe

Everyone needs to network and connect… we need to do this across all countries to get a better global deal for all

instability in Europe and the World, crisis and maybe war:-(

#### ICT professionals

ICT was categorized as “a key profession in this century” with many comments noting the value of ICT professionals in a range of ways (102). These included supporting new business models (39), ICT networks (22), innovation and change (38), new technologies (39) and decision making (11). There was a strong sentiment that ICT professionals would be needed to build and develop new systems supporting national change regardless of the Brexit position, but this had further highlighted society’s information needs in a wide range of ways. In addition, the existence of an ICT profession with supporting professional bodies was seen to deliver frameworks and standards which promote ethical behaviors. These overarching professional values and networks were seen to have the capacity to transcend national boundaries. This in turn was seen as an opportunity for stronger information governance for society and the individual citizen.

#### Information needs

Information needs, in a variety of forms, were coded 202 times across all factors. A number of the comments from participants within and outside the UK focused on the need to understand the Brexit Leave vote and to reflect on this decision and its implications in an informed and wide ranging manner. The Brexit political campaigns, as well as the media and social media coverage, were noted to have raised genuine issues about the need for ‘honest’ and ‘truthful’ information to be available to all, in order to enable a democratic decision making process. Not only was information noted not to have been available but, furthermore, it was perceived that there had been misinformation. Comments included:

There is a great deal of uncertainty at the moment and people need somewhere where they can get trusted information.

An increased recognition of the need to verify information used to underpin democratic debate as part of the democratic process; and to encourage all areas of society to value—and demand—such information.

Social media was felt to have failed in providing channels for genuine debate. One participant noted that there had been an actively “destructive use of social media” with another stating:

The Brexit campaign has demonstrated that many dominant information channels (traditional and social media) cannot be relied upon to give unbiased, accurate information.

In response to these challenges, it was clearly perceived that ICT professionals could assist with addressing these failings not only in terms of the democratic process but also to support information provision and scrutiny for wider societal concerns. The Brexit campaign highlighted information literacy needs (17) which could be addressed in schools, HEFE and through libraries. Clearly identified was the need for ICT channels and content which would be trusted and was accurate (11), unbiased (9), up-to-date (6), and open to interrogation. Organizations were identified as needing to provide transparency in terms of delivering this data/information (16). This was needed to address future events related to Brexit and beyond.

Well, it turns out that over half the country has basically no information literacy or critical thinking skills (including the idiots who didn't vote)

Public libraries could have a role in providing accurate, up-to-date and easily understandable information to the public.

Requirement for high-quality, balanced and straightforward information to navigate these incredibly uncertain times!

In terms of delivering Brexit, information and ICT tools were highlighted as essential for “scaffolding the delivery of any Brexit change” whether in terms of providing information for decision making or for disentangling EU legal processes or data analytics to advance economic models or trade positions; in essence to “unpack and separate EU systems and build new ones”. It was noted that in moving out of the EU, the UK could lose access to shared data and storage systems which help keep data safe and also provide key information for a range of UK needs, including security and business; e.g. two people noted that the insurance industry relies on EU claims data. This would need to be addressed and potentially sourced in new ways taking advantage of the fluid boundaries of ICT delivery. Participants recognized the opportunities for information and ICT professionals:

Info & ICT professionals will be needed to help work through the complex business implications of Brexit and there is significant potential for job growth in this area.

Highlighting the need for good information management which will be required to separate EU and UK legislation and policy.

There will likely be an increased need for accurate, up-to-date, easily understandable and accessible information for different groups of UK citizens/residents about how Brexit affects areas of their everyday lives (e.g. employment law, equality, pensions, benefits, access to health services, international travel, business regulations, consumer protection etc).

#### Information rights law

The UK move away from the EU was seen as enabling the UK to deliver new bespoke laws more quickly and in particular to respond to digital opportunities and needs. Information rights laws were identified “as invaluable pillars of a democratic society” providing information access rights, intellectual property rights including copyright, and the protection of personal data. There was a strong focus on the latter protections as the General Data Protection Regulation (GDPR) comes into force across the EU in May 2018 [38]. The Regulation was passed by the EU in April 2016 after several years of negotiations. It marks a significant change in managing personal data and protecting the privacy rights of EU citizen. The potential loss of GDPR as part of UK law was seen by many as a threat to the personal data rights of UK citizens. It was noted that the transfer of EU personal data for trading purposes relies on GDPR ‘adequacy’, and therefore a majority (99) identified continued compliance as an essential opportunity for the UK. 91 comments noted that Brexit presented a chance to alter GDPR and deliver a system which could deliver benefits including greater data exchange with nations outside the EU.

In addition, other aspects of the law were noted as needing to be addressed globally given the concept that information can flow across national borders dependent upon ideas around the desirability of data sharing and data portability. Overall, information borders across nations were seen as undesirable (50) and legislation was seen as key in terms of ensuring open border or boundaries for information flow.

Interestingly lawyers were the only profession which was discussed negatively, with the Brexit process being noted as providing more jobs for lawyers in order to negotiate and legislate through the process across the EU. This was perceived by some as a waste of public monies (9).

#### ICT innovation

ICT was seen to be at the heart of driving innovation and supporting needs for citizens, trade and governments across the EU and globally including developing new global supply chains reaching new markets (92) and new networks (43) and underpinned by new business models (39). Linked to driving innovation and change (38) was the development of new software (26), the facilitation of connections through ICT and Cloud networks (22), data analysis with data analytics (18) and data exchange and portability (14). A range of tools were highlighted as valuable for these processes including semantic technologies, data analytic software and data portability mechanisms. ICT was distinguished as a great opportunity for those who could engage with change as it can often be harnessed in agile and complex ways to engage new markets. In this context, Brexit was recognized as being motivational (21) causing people to look at things in new ways and pushing new boundaries. Comments included that it would push ICT professionals and practice to:

Engage globally

Open us to innovative approaches across the whole world

Increase entrepreneurial spirit when professionals and their orgs have to look for opportunities further afield than EU.

We can help the UK strategise–as tech has the tools–and reconfigure operations. Tech isn’t land based and it can navigate boundaries and seamlessly cross frontiers. We can help with this so I hope that whilst the industry will shift the UK will continue to be known as an area of tech expertise

## Conclusions

Immediately after the Brexit decision the research reveals uncertainties amongst ICT professionals regarding what the decision would mean, with just under half of the participants not identifying any opportunities or threats. However, for those who did, threats outweighed opportunities by just more than double. This is perhaps not unexpected given 76% of the participants were negative about the vote to leave. Whilst understanding the global possibilities and dangers, participants saw their position from national and organizational perspectives. Despite making the case for the opportunities delivered by increased global connection(s) these parochial concerns were more evident.

The highest frequency coded threats related to areas outside the participants’ control and the highest frequency opportunities related to areas where there was the potential for ICT intervention and control. This is aligned to research which indicates that humans are hardwired to react negatively to uncertainty and the ability to control a situation will minimize stress [39]. The threats identified were wide ranging with key examples from the individual STEEPLE factors including:

- Social-cultural concerns relating to closed cultures, xenophobia, diminished networks, uncertainty, loss of human rights including information rights, and cuts impacting most heavily on the most vulnerable in society.
- Technological concerns relating to information borders, loss of ICT skills, networks and trade, and loss of standards, security and information rights laws.
- Economic concerns related to uncertainty and conditions that could foster a recession and cuts with wide ranging impacts.
- Environmental concerns including loss of shared global environmental agendas, standards and laws, resources and moves away from renewable and sustainable energy supplies.
- Political concerns relating to national introversion, moves to extreme politics and loss of socialist policies.
- Legal concerns including loss of unified information rights laws including copyright, access to information, media law and personal data protection as well as the systems delivered through the standards of EU social policies, laws and courts.
- Ethical concerns including loss of policies, standards, laws and resources diminishing ethical priorities and potentially fostering conditions for bribery and corruption.

The strongest themes in regards to opportunities were linked to factual data and possibilities related to the more substantively available information. For example, information needs around the perceived failing of accurate trustworthy data provided during the Brexit campaign were seen to be clear opportunities. The participants drew upon ICT professional knowledge sets, such as information literacy, in order to suggest ways to support society moving forward. Other opportunities were around a known piece of EU legislation (GDPR), ICT networks, business models and global supply chains for goods and services. The opportunities identified contained wide ranging possibilities with key examples from the individual STEEPLE factors including:

- Social-cultural possibilities relating to a clearly identified set of information needs to better support society including the provision of information literacy education and the provision of accurate, truthful and up-to-date information for a wide range of needs. In addition, the Brexit decision was noted to provide the potential to reassess national boundaries, networks and movement, not only from a UK and EU perspective but also globally.
- Technological possibilities relating to the need for innovation in times of change and the possibility of looking to new networks and developing new laws to deal with ICT developments.
- Economic possibilities to develop new global networks, markets and trade models.
- Environmental possibilities to develop a focus on community action, new networks, digital supply chains, shorter supply chains and localized markets.
- Political possibilities relating to new global networks, a renewed focus on democracy, open accurate trustworthy data and citizen needs.
- Legal possibilities to develop bespoke digital laws, greater harmonized information rights laws and quicker legislative processes.
- Ethical possibilities relating to global networks and a greater emphasis on governance and accountability.

The factors are not mutually exclusive and as the above examples highlight there are connections between the opportunities and threats across the seven factors.

The survey is part of a longitudinal piece of research. Using the same methodological approach two further surveys are planned. The second will be one year after Article 50 was triggered on 29 March 2017. The final survey will be one year after the UK exit from the EU, assuming this occurs. This will enable the response to change to be charted as the transformation delivered by the Brexit vote becomes clearer. This approach will reveal whether the initial uncertainties and negative reactions revealed relating to the change diminish over time charted against the different STEEPLE factors and what the perspectives and responses are to the challenges and opportunities that arise at key points.

## Supporting information

S1 Fig (PDF)

S1 Table (XLS)

S2 Table (XLS)

S3 Table (XLS)

S4 Table (DOCX)
