# Supplementary material for: Engaging with change: Information and communication technology professionals’ perspectives on change in the context of the ‘Brexit’ vote
Source: PLoS One. 2017 Nov 8;12(11):e0186452. doi: 10.1371/journal.pone.0186452 (PMC5695584; doi:10.1371/journal.pone.0186452)
Supplement: S1 Fig — (PDF) [file pone.0186452.s001.pdf]

Research overview

Brexit presents a significant change for the UK. However, those professionals who work in the Information Communication and Technology (ICT) sector and/or manage information, whether in the public or private sector, are well practised at responding to fast changing environments to harness opportunities.

This survey aims to capture perspectives on the opportunities and threats that face those delivering information agendas across the UK. We hope to identify ideas that information professionals can harness to support the UK changing landscape and also flag areas of information management/ICT weakness in the light of Brexit.

The survey is open to anyone. Although the focus is on the UK position we are keen to hear where those outside the UK see opportunities to collaborate with the UK or have reservations about engaging with the UK in the light of Brexit. We hope people will contribute in a constructive manner even when noting concerns. We aim to publish the results quickly in an open access journal and plan to repeat the study at the point of Brexit and two years thereafter.

The survey is anonymous and we are not collecting IP addresses. We do ask for you to log your place of residence and your nationality for demographic purposes. The information you provide will be retained securely and anonymously and will be kept in line with University research data management and retention policies.

There are 20 questions (all optional). The survey will take 10-20 minutes to complete depending on how much you wish to write. There are no right or wrong answers to any of the questions.

We know that you are busy and appreciate your time. You can exit the survey and your previous comments will be saved so that you can go back and complete it when you have time. The survey will close on Monday 25th July at 4pm.

The research is a partnership between the iSchools at UCL (Dr Elizabeth Lomas) and Northumbria University (Professor Julie McLeod). The survey is being facilitated by Dr Elizabeth Lomas e.lomas@ucl.ac.uk

Thank you for your help.

1. In regards to Brexit generally, do you feel...?

- Positive
- Negative
- Both positive and negative
- Neutral
- Not sure

2. Do you believe that Brexit is...?

- An opportunity for information and ICT professionals
- A threat for information and ICT professionals
- A combination of opportunities and threats
- Too unpredictable/complicated to make a judgement
- Not sure

3. In which region do you reside?

4. Which is the country/region that most closely defines your nationality?

5. What is your age?

- 18 to 24
- 25 to 34
- 35 to 44
- 45 to 54
- 55 to 64
- 65 to 74
- 75 or older

6. What is your current occupational status? (You may tick more than one option)

- [ ] Employee
- [ ] Self-employed
- [ ] Unemployed
- [ ] Student
- [ ] Volunteer
- [ ] Retired
- [ ] Other (please specify)

7. Which best describes your profession/ job function/ area of study? (You may tick more than one option)

- [ ] Academic
- [ ] Administrator
- [ ] Archivist
- [ ] Business analyst
- [ ] Cyber security
- [ ] Data manager
- [ ] Information manager
- [ ] IT support
- [ ] Information security expert
- [ ] Lawyer
- [ ] Librarian
- [ ] Management consultant
- [ ] Marketing manager
- [ ] Mergers and Acquisitions expert
- [ ] Records manager
- [ ] Software developer
- [ ] Web designer
- [ ] Managing Director
- [ ] Manager
- [ ] Consultant
- [ ] Project manager
- [ ] Other (please specify together with your job title)

What is your job title?

8. If working/volunteering within which sector would you place your organization?

Other (please specify) or add additional information

9. Approximately how many employees are there within your organisation?

- 0-9
- 10-49
- 50-250
- 250-1,000
- 1,000-10,000
- 10,000+

10. Do you manage any staff?

- Yes
- No

11. If you are working or volunteering is your organisation a global entity operating from sites spread across the World?

- Yes
- No

12. Does your organisation have any plans in the light of Brexit?

- Yes
- No
- Don't know
- Are you able to briefly outline what these are?

Opportunities and Threats

Taking a 'STEEPLE' model this final page seeks to gather your ideas on opportunities and threats for information and ICT professionals in the context of the UK Brexit decision. Against each domain, from within the STEEPLE model please record your ideas for Leave opportunities or threats. If you cannot think of an opportunity or threat then leave the fields blank. There is a chance to record any further thoughts in the last question.

13. Socio-cultural factors (S) may include customs, lifestyles, and values that characterize the society in which an organization/individual is operating. They can also include demographics of age, population growth rates, level of education, distribution of wealth, social classes and living conditions. Socio-cultural factors may influence entrepreneurial spirit, fashions and consumer demands, the ability of a society to obtain resources or trade in certain ways due to consumer influence.

Can you identify a socio-cultural opportunity and/or a threat for information/ICT professionals in the UK in the light of Brexit? [Leave fields blank where you do not have any ideas]

Opportunity

How could this opportunity be harnessed?

Threat

How could this threat be avoided/minimised?

14. Technological factors (T) refer to the rate of new inventions, development and changes in technology including software, hardware, networks and e-commerce. They can also include: methods of manufacture, distribution and logistics, attitudes to research and research spending.

Can you identify a technological opportunity and/or a threat for information/ICT professionals in the UK in the light of Brexit? [Leave fields blank where you do not have any ideas]

Opportunity

How could this opportunity be harnessed?

Threat

How could this threat be avoided/minimised?

Economic and Environmental Factors
Please add any opportunities or threats. Leave blank and move on to the next questions if you have nothing to add

15. Economic factors (E) represent the wider economy so may include: sector growth, levels of employment, consumer confidence, costs to the sector (e.g. hardware and licences), interest rates and monetary policies, exchange rates, inflation, investment opportunities, research funding etc.

Can you identify an economic opportunity and/or a threat for information/ICT professionals in the UK in the light of Brexit?

Opportunity

How could this opportunity be harnessed?

Threat

How could this threat be avoided/minimised?

16. Environmental factors (E) include energy and resource-efficient goods (carbon neutral, recycling etc) services and technologies and promotion of informed choices by customers. They also include threats from natural disasters.

Can you identify an environmental opportunity and/or a threat for information/ICT professionals in the UK in the light of Brexit? [Leave fields blank where you do not have any ideas]

Opportunity

How could this opportunity be harnessed?

Threat

How could this threat be avoided/minimised?

Political and Legal Factors
Please add any opportunities or threats. Leave blank and move on to the next questions if you have nothing to add

17. Political factors (P) refer to the stability of the political environment and the approaches of political parties, sector interest groups and other stakeholders. This may include: policies on tax, trade or the sector in general. Political factors are related to Legal factors (L). The difference between Political and Legal factors is that Political refers to the approach of political actors, whereas Legal factors refer to actual legislation. Legal needs to be implemented whereas Political may represent influence, restrictions or opportunities.

Can you identify a political opportunity and/or a threat for information/ICT professionals in the UK in the light of Brexit? [Leave fields blank where you do not have any ideas]

Opportunity	
How could this opportunity be harnessed?	
Threat	
How could this threat be avoided/minimised?	

18. Legal factors (L) such as: domestic legislation, EU regulation, self-regulation, standard agreements, international trade agreements, national and international competition law, trade union agreements and consumer protection. Brexit will mean that the UK can rethink compliance with EU legislation but what does this mean for information/ICT opportunities or threats?

Can you identify a legal opportunity and/or a threat for information/ICT professionals in the UK in the light of Brexit? [Leave fields blank where you do not have any ideas]

Opportunity	
How could this opportunity be harnessed?	
Threat	
How could this threat be avoided/minimised?	

Ethical Factors

Please add any opportunities or threats. Leave blank and move on to the next questions if you have nothing to add

19. Ethical factors (E) include the moral expectations determining how individuals and organisations should operate. This is the domain of governance which extends beyond the strictures of law and influences approaches to accountability and confidentiality, bribery, intellectual property rights, brand and reputation.

Can you identify an ethical opportunity and/or a threat for information/ICT professionals in the UK in the light of Brexit? [Leave fields blank where you do not have any ideas]

Opportunity	
How could this opportunity be harnessed?	
Threat	
How could this threat be avoided/minimised?	

20. Do you have any other comments that you wish to add?
