# Supplementary material for: Engaging with change: Information and communication technology professionals’ perspectives on change in the context of the ‘Brexit’ vote
Source: PLoS One. 2017 Nov 8;12(11):e0186452. doi: 10.1371/journal.pone.0186452 (PMC5695584; doi:10.1371/journal.pone.0186452)
Supplement: S4 Table — (DOCX) [file pone.0186452.s005.docx]

S5 Table Sample of responses

| 1.0 | SOCIAL-CULTURAL FACTORS |
| --- | --- |
|  | Sample quotes - social-cultural opportunities |
| 1.1 | to renew and develop worldwide and individual country relationships. |
| 1.2 | Increased opportunities for joint projects, job exchanges, visits, with professional colleagues outwith Europe. |
| 1.3 | Greater impetus to develop the UK workforce |
| 1.4 | Study change as a divestiture which is not well established in the IS literature |
| 1.5 | Need to utilise more the foreign-language skills and cultural know-how of staff |
| 1.6 | Re-establish British culture, which is resilient, disciplined, diplomatic, hardworking and mature |
| 1.7 | None |
| 1.8 | Kick up the bum |
| 1.9 | Extend existing and develop further links with partner organisations and diasporal students worldwide |
| 1.10 | Collaboration with like-minded souls across the world |
|  | Sample quotes – harnessing social-cultural opportunities |
| 1.11 | From Australia would look at greater corporation in Intelligence gathering, big data sharing |
| 1.12 | UK could harness the opportunity to market its history, culture and nature - an opportunity for cultural and heritage institutions |
| 1.13 | People clearly need develop their information seeking skills, also skills around finding reliable information and critical thinking. |
| 1.14 | More business through deregulation |
| 1.15 | Development of what we want as a nation |
| 1.16 | Using networks such as LinkedIN, various social media tools, and developing personal, informal networks and contacts. |
| 1.17 | Boost (secondary and) higher education standards |
| 1.18 | Positive and active foreign policy from government and every industry |
| 1.19 | Not leaving the EU |
| 1.20 | forming closer ties between UK customers across the various sectors to develop more understanding among suppliers. |
|  | Sample quotes – social-cultural threats |
| 1.21 | General concern that Brexit represents a slide towards quite extreme rightwing politics, possibly also to be reflected in a potential Donald Trump presidency. Initial increase in racially motivated hate crimes reinforce this fear, and may suggest a move towards a more 'closed' or insular UK. This climate is fundamentally inhibiting for the free flow of information and worrying for a great many individual information professionals. |
| 1.22 | Loss of employment due to economic downturn |
| 1.23 | Data Protection Act is disrupted- Artcile 8 |
| 1.24 | Left out of the conversations where funding has been strong |
| 1.25 | When global funding opportunities become more scarce, which they will when we leave Europe, and a government that doesn't support Libraries continues to slash our budget, the Library must necessarily make up the difference and will start acting more like a commercial enterprise rather than social enterprise. The first thing to go is free access to collection items (particularly digitised items: we'll have to sign up with more corporations to digitise the collection at the expense of an embargo on the content) and public programming (such as exhibitions and events which have the power to expose the masses to new ideas and cultures....but will now be charged at a rate only accessible to those who can afford it, and arguably benefit from it less.) |
| 1.26 | I work for company that receives a large number of grants from European bodies for research. If favourable deal cannot be reached I can envisage the business incorporating elsewhere in Europe as is only right and proper in order to continue to maximise research grants and opportunities. |
| 1.27 | Some integrated (or joined-up) information may become disintegrated or perhaps more fragmented. A retreat into nationalism,regionalism, parochialism within UK and the undoubted effects that might have on how people view information and its ownership - information might conceivably flourish |
| 1.28 | Less diverse employees |
| 1.29 | Growing xenophobia |
| 1.30 | Long term uncertainty |
|  | Sample quotes – minimizing social-cultural threats |
| 1.31 | guarantee rights of EU citizens, enable free movement |
| 1.32 | Focus on the achievable |
| 1.33 | By spelling out the consequences of a brain drain to the new May government so that Brexit can be minimalised |
| 1.34 | A lot of expensive lobbying |
| 1.35 | Keep international information sources and supply open |
| 1.36 | Continue to welcome foreigners to the UK (as students and workers); continue to work with EU countries. |
| 1.37 | If the government decides to keep EU nationals already living in the UK since i am a french citizen |
| 1.38 | Global outreach at all stages |
| 1.39 | Pressure on government not to trigger article 50 |
| 1.40 | Guarantee right to remain for EU citizens currently in the UK; guarantee principle of free movement |
| 2.0 | TECHNOLOGICAL FACTORS |
|  | Sample quotes – technological opportunities |
| 2.1 | Developing the idea of 'Libraries without walls', on a global basis |
| 2.2 | ICT need not be bound by nations or regions it can transcend these physical limitations |
| 2.3 | Particularly in the software domain there could be opportunities. Many small and middle-sized business need to get back to dealing with trading barriers (e.g. tolls) that new software could support. |
| 2.4 | Again a more international outlook on work and research for the profession can be a benefit |
| 2.5 | UK might now skip - at least in part - the so-called "precautionary principle" adopted by EU: if this principle had been adopted, possibly modern technology would have nevere developed |
| 2.6 | ICT solutions (e.g. EDRMS that easily comply with data portability requirements for businesses |
| 2.7 | We can in ICT think strategically at both a high level about mapping our connections and collaborations and then looking at the granular detail of the where and how of delivery |
| 2.8 | UK could perhaps fully follow more streamlined US approaches to IM |
| 2.9 | More freedom to develop new products and services without having to comply with strict EU regulations |
| 2.10 | Change is motivational esp in tech where can be adaptable |
|  | Sample quotes – harnessing technological opportunities |
| 2.11 | Prioritisation of start-up funding and grants for new businesses; government funding priorities to kick-start specific industries |
| 2.12 | Invest in the UK Universities and research. |
| 2.13 | Map out the opportunities we know exist for thinking about harnessing the best ICT laws |
| 2.14 | Seeking new parternships with Commonwelath countries (e.g India) , USA, etc. |
| 2.15 | Serious commitment from the government to invest in digital infrastructures to improve access for all both directly and through funding the necessary research required for harnessing and implementing new technologies. |
| 2.16 | Agreement between institutions RE making research open and transparent, no borders. |
| 2.17 | The EU does limit data use storage competition etc so now the message can be sold that tech is more agile in the UK the media can assist with this |
| 2.18 | UK adopt all EU laws as UK laws in this area but immediately seek to modify to our needs with input of technological staff rather than corporate folk with no in depth understanding of issues and opportunities. |
| 2.19 | Keep data protection and GDPR for data flow reasons as well as personal data rights |
| 2.20 | None |
|  | Sample quotes – technological threats |
| 2.21 | we could fall behind standards. Reduced stimulation from technology immigrants slows development |
| 2.22 | Data handling laws no longer governed by eu |
| 2.23 | Diminished access to projects/work/funding within EU states as EU funding diverted from UK |
| 2.24 | EU links and lost expertise , eg Poles are key part of ICT workforce |
| 2.25 | If EU data laws (e.g. Data Protection legislation) are no longer applied, there is a real threat to how businesses might then handle personal data; it will also be costly to renegotiate data protection (for example) terms in software contracts. |
| 2.26 | tarriffs on software, servers in UK with different regulations |
| 2.27 | The implications of having different data protection laws in the UK to the EU is potentially damaging to cloud based computing. The UK as a hub for ICT development is also under threat if the free movement of goods and services are not preserved/ |
| 2.28 | Gathering information from international online platforms might come with more impediments |
| 2.29 | Loss of standards and loss of input into development on standardisation |
| 2.30 | Loss of research funding for UK universities |
|  | Sample quotes – minimizing technological threats |
| 2.31 | In the Brexit negotiations; by finding EU partners and cooperating in the new trade frameworks. |
| 2.32 | Replicating eu law |
| 2.33 | Wide-scale befriending campaign and enthusiastic participation in existing technological partnerships |
| 2.34 | Ensuring data portability both technically, legally and through networks. |
| 2.35 | Strengthen tech education in schools and beyond |
| 2.36 | Develop a clear research investment agenda which reaches out for new collaborations |
| 2.37 | Welcome our new Indian and Chinese overlords? |
| 2.38 | Improve UK maths, science & engineering education and status. |
| 2.39 | Risk management for each aspect of IT threats |
| 2.40 | Keep GDPR |
| 3.0 | ECONOMIC FACTORS |
|  | Sample quotes – economic opportunities |
| 3.1 | world trade |
| 3.2 | Won’t be bound by same competitions laws |
| 3.3 | Worldwide reintegration. |
| 3.4 | For now I cannot see any economic benefits to Brexit..... maybe a weak pound could lead to dollar investment |
| 3.5 | Provide services to support new management strucutres |
| 3.6 | If the Brexit does not backfire on the UK, or if the UK economy manages to recover from its effects, and more freedom of market actually boosts the economic growth, that coould actually benefit all the sectors in the society, both public and private. |
| 3.7 | Interest rates may rise, meaning independent funding bodies will have more income |
| 3.8 | Stronger Euro |
| 3.9 | Banks go to Frankfurt/Main |
| 3.10 | New tolls and global connections |
|  | Sample quotes – harnessing economic opportunities |
| 3.11 | Maximise use of the exchange rate fluctuations |
| 3.12 | The creation a national programme of industrial investment with particular attention on our information based industries. |
| 3.13 | Tax breaks to make UK competitive. Look at pound to seize advantage |
| 3.14 | While this example of taxation in a single area is an opportunity for the EU, I feel that the government will still maintain the revenue which such a tax would bring in by levying an alternative tax (either via indirect or direct taxation) |
| 3.15 | Connection new globally |
| 3.16 | As IT is a growing and changing industry, entrepreneurial companies should thrive - i.e. innovate and sell. |
| 3.17 | Mobilising communities to fight against austerity, demand access to free education and renationalise public services. |
| 3.18 | New networks and trade models driven by ICT |
| 3.19 | We will need to look around at the markets |
| 3.20 | At the moment, the economic future is forecast in somewhat bleak terms, so if there are positives, these need to be highlighted more; more links with global partners need to be established |
|  | Sample quotes – economic threats |
| 3.21 | Loss of EU regional project funding for capital and education funding and the jobs that accompany it. Loss of inward investment in IT systems from the US/NAFTA and other external economic areas |
| 3.22 | Economic shrinking, isolationism |
| 3.23 | UK shut out of the single market |
| 3.24 | Uncertainty |
| 3.25 | Falling pound is already having an effect on information services, dramatically increasing costs of subscriptions paid in Euros or dollars. Likely to be further cuts on public services, which may well hurt information services. |
| 3.26 | Economic downturn which could lead to job cuts. Archivist jobs are often seen as a bit of a 'luxury' so tend to be cut when things are not so good financially. |
| 3.27 | Recession caused by no longer having access to the single market |
| 3.28 | Less fund in the Higher Sector, means less research, less need for employees |
| 3.29 | Interest rates and weak £ |
| 3.30 | According to the BBC, the guardian, the economist etc. we should expect some negative impact on the UKs economy (slow growth or even small recession) accompanied with loss of employment and all the things mentioned above with a negative connotation. |
|  | Sample quotes – minimizing economic threats |
| 3.31 | Continued enthusiastic participation in thye European Economic Area and early notification that our EEA participation will continue beyond 2018. Strong line that Article 50 will not be activated until we are certain of the type of Brexit we want; not giving way to thye early activation seemingly favoured by the Juncker faction in the Commission. |
| 3.32 | Work smarter |
| 3.33 | Get trade deal with EU look at new markets |
| 3.34 | Move to Canada? |
| 3.35 | New immigration will create stronger skills pool and better trading support or innovation etc |
| 3.36 | Try to make sterling more buoyant; speak to an Economist about how to do that. Anti-austerity seems a good start. |
| 3.37 | Re-Brexit |
| 3.38 | stay in the single market |
| 3.39 | Who knows the answer to that question?! |
| 3.40 | Finding alternative sources of funding (although those are extremely few and far between) |
| 4.0 | ENVIRONTMENTAL FACTORS |
|  | Sample quotes – environmental opportunities |
| 4.1 | we could focus more on specific island/UK issues |
| 4.2 | reduce environmental 'red tape' saving staff time and cost |
| 4.3 | None |
| 4.4 | The creation of regulatory environment more suited to UK conditions. |
| 4.5 | to enhance local laws |
| 4.6 | Digitisation - Digital Records never traverse to Paper |
| 4.7 | Even if EU has possibly the best environmental legislation in the world, its disorder may often create just more chaos: UK might harness a more straighforward process to set up its environmental rules |
| 4.8 | Global focus |
| 4.9 | Research for power |
| 4.10 | Digital is part of the environmental solutions |
|  | Sample quotes – harnessing environmental opportunities |
| 4.11 | information and data vital for measuring progress and innovation. |
| 4.12 | Smart tools to better visualize and manage resources |
| 4.13 | By realising that the basic concepts underpinning EU environmental action were good, far better that those of China, USA and Russia |
| 4.14 | Software/Hardware |
| 4.15 | If more companies more out of GB, the environmental footprint will be smaller compared to now |
| 4.16 | It is vital to have a global focus if we are to actually tackle all the many environmental issues. Maybe focusing beyond EU will help. |
| 4.17 | New environmental laws |
| 4.18 | Look at digital solutions |
| 4.19 | Require hosting within jurisdiction in regulations |
| 4.20 | Set up a recycling company to deal with this |
|  | Sample quotes – environmental threats |
| 4.21 | Risk that preoccupation with Brexit, and declining national finances will leave UK unable to develop resilient and sustainable approaches to tackling climate change. Any infrastructure investment is likely to go on big prestige projects (eg HS2) - there is unlikely to be anything left to fund information infrastructure. |
| 4.22 | Worsening of the environmental situation, decrease in sustainability |
| 4.23 | Reduced legislation in this area leads to increased environmental impact of ICT services |
| 4.24 | Isolation when this is a global issue |
| 4.25 | Research which by its very nature needs to cross borders (because it's dealing with global issues) will be hampered |
| 4.26 | The creation of diluted standards in order to meet short term requirements of supporting economic growth. |
| 4.27 | Of course, the fact that leaving EU may result in a worse level of awareness as far as environemntal themes are concerned |
| 4.28 | Lack of accountability |
| 4.29 | less push for green power |
| 4.30 | No money for the environment if the global economy collapses |
|  | Sample quotes – minimizing environmental threats |
| 4.31 | Open data |
| 4.32 | Campaigning; implementing the "Internet of things" (for environmental monitoring and evidence gathering). |
| 4.33 | Voluntary code of practice |
| 4.34 | Legislate |
| 4.35 | Digitisation Strategy |
| 4.36 | Don't leave the EU |
| 4.37 | Have a global agenda |
| 4.38 | Again, if Britain wants to remain part of the single market it would obviously need to have its environmental laws harmonised with Europes. I can't see how such fundemental legislative function can be repatriated to Westminster if the UK wants to remain in the single market |
| 4.39 | A referendum is only an opinion poll - it does not have to be accepted. Lobby the government to instead solve the problems that have been highlighted by both sides of the argument and halt Brexit. |
| 4.40 | Education and awareness. |
| 5.0 | POLITICAL FACTORS |
|  | Sample quotes – political opportunities |
| 5.1 | It will give our Government the chance to rule Britain not a foreign nation |
| 5.2 | Restore democracy |
| 5.3 | Shift in geo-political focus. Australia and the UK have a lot in common, the EU was an impediment to Australian interests. |
| 5.4 | Move left! |
| 5.5 | Too soon to tell |
| 5.6 | Clearly there is a very disgruntled electorate that cuts across several divisions. Now that this has been exposed, it is an opportunity for politicians to try to understand the cause of this and rethink their policies to change the way people think about themselves and their identity and worth. |
| 5.7 | Open Government |
| 5.8 | influence on localised policy |
| 5.9 | We can carp on about the lack of critical thinking skills in the British public |
| 5.10 | General Election. Progressive parties enter into a coalition. Tories & UKIP are electorally wiped out. |
|  | Sample quotes – harnessing political opportunities |
| 5.11 | develop and maintain worldwide and country relationships. Work hard on understanding & diplomacy |
| 5.12 | Directly elected EU Commission (with safeguards to ensure representation from all member states); bigger role for EU Parliament. |
| 5.13 | Taking a non-sectarian view of the situation and respecting the view of the majority within NI |
| 5.14 | Re-positioning of talents available at the UK Office of the European Community to be directed towards other international bodies and groupings |
| 5.15 | A constitutional convention and possibly the creation of more formally federal state in recognition of increasing political diversity within the home nations of England, Scotland, Wlaes and Northern Ireland. |
| 5.16 | Lobbying for joined up policies to support IT |
| 5.17 | More democratic government to be elected |
| 5.18 | Research, higher education and training |
| 5.19 | Building on existing trade agreements within the Commonwealth and with the US |
| 5.20 | M professionals first need to articulate their requirements / case |
|  | Sample quotes – political threats |
| 5.21 | Huge political uncertainty, policy vacuum until the negotiating position is clearer. Likely to be even more uncertainty during the negotiations and when UK finally exits. |
| 5.22 | UK will become more protectionist; UK politics may become more fragmented. |
| 5.23 | Trusting UK companies and Politics and trusting long term agreements. |
| 5.24 | Become an undemocratic and entirely marginal entity within an "English" UK |
| 5.25 | Loss of friends/contact who succumb to xenophobic literature / propaganda put pout by anti-EU bodies |
| 5.26 | No changes to the current situation. |
| 5.27 | That Britain looks like a small-minded, racist, little island, and bad feeling is increased between locals and foreigners (including British born of parents of other nationalities). |
| 5.28 | political isolation, difficult and disadvantageous contracts |
| 5.29 | Might EU companies with offices in the UK move out? |
| 5.30 | Loads |
|  | Sample quotes – minimizing political threats |
| 5.31 | Negotiate free-trade agreements (and accept the conditinos attached); work with the main parties to agree pragmatic policies (for Government AND Opposition!) |
| 5.32 | Counter-action |
| 5.33 | think it would be good for more people to become activists. We need more library groups, more student and staff groups, greater union involvement, generally, we need more people speaking out against the threats caused by Brexit. |
| 5.34 | Change recommendation algorithms so that more unlikely hits occur in the result set. E.g., instead of reading only about the leave campaign, recommendations by Google, Facebook etc.. also suggest info from the pro campaign. Move to an "internet of truth" in order to provide a clearer picture, we should not only focus on gathering data but on gathering and linking actual facts (there exists a really good Zeit.de article on the topic of data vs. facts) |
| 5.35 | Tackle the economic inequality and provide adequate public services in communites as these issues if ignored fuel the far right |
| 5.36 | Punish any cases of discrimination |
| 5.37 | Focus on making positive connections around a global trade focus. Make day to day transactions a good thing. |
| 5.38 | Unclear. |
| 5.39 | Clear guidlines, sooner rather than later |
| 5.40 | Widening the viewpoint and avoiding local politicians' intensive control |
| 3.0 | LEGAL FACTORS |
|  | Sample quotes – legal opportunities |
| 6.1 | Get rid of the European Driving regulation and then professional will be able to travel and work to their full potential. |
| 6.2 | Get rid of FOI legislation that has had a detrimental effect on recording key decision making. |
| 6.3 | To harmonise data protection adn confidentiality laws |
| 6.4 | Able to rewrite rules to give access to non-EU resources |
| 6.5 | Creating laws encouraging better record-keeping & data protection measures |
| 6.6 | Too soon to tell. UK is relatively lightly regulated |
| 6.7 | Keep GDPR |
| 6.8 | Laws that reflect the digital environment and continue to be updated quickly |
| 6.9 | Improvements to working time directives |
| 6.10 | Still need to comply with EU GDPR and existing EU information related legislation |
|  | Sample quotes – harnessing legal opportunities |
| 6.11 | Understnad the new laws and offer advice and software tools to multinational companies and companies that wish to operate across borders. |
| 6.12 | ICT research into those areas and lobbying for laws sketched out and focused on providing support to high interest aims of the UK |
| 6.13 | The UK could create more sensible copyright laws |
| 6.14 | Working with global companies in the developing as well as the developed world. |
| 6.15 | Protectionist policies and tariffs |
| 6.16 | Review all data laws don't just automatically mimic EU but think through issues with top tech experts not just lawyers |
| 6.17 | GDPR |
| 6.18 | Keep in close touch with relevant cross-Europe organisations |
| 6.19 | Keep EU laws |
| 6.20 | None |
|  | Sample quotes – legal threats |
| 6.21 | Loss of ability to provide services to EU countries based on common acceptable data protection policies. Danger of less employee protections, environmental protections, consumer protections and possible "race to the bottom" of deregulation. |
| 6.22 | Unnecessary and unwanted complications in administering European-wide best practice on issues such as copyright and IPR |
| 6.23 | Loss of influence on the drafting of EU legilsation. |
| 6.24 | If we are to continue trading with the EU we will need to continue to match EU legislation but we have given up our rights to be involved in negotiations for such legislation. For example if we are to continue to trade effectively with the EU the UK will still have to adopt the GDPR to replace the Data Protection Act - interpretation of the new Regulations will no longer be in our hands but imposed by EU if we are to continue to trade. It will add more complexity to decisions regarding cloud based storage using US server farms for example because the UK will not formally be covered by the US/EU Privacy Shield arrangements and will have to arrange something bilaterally (whilst still staying the right side of EU GDPR) |
| 6.25 | If UK doesn't meet new GDPR requirements then business may go elsewhere as the UK would not meet the adequacy assessment |
| 6.26 | Lots - the EU has developed a wide range of very good pieces of legislation to improve our society including ensuring safe working environments, minimum wage, equal rights, human rights, data protection - the list goes on. |
| 6.27 | Unpicking of EU influenced legislation could provide opportunity for law makers to weaken legislation regarding FOI, IPR etc |
| 6.28 | Lack of standardization with other EU countries on important ICT issues |
| 6.29 | Open Access to articles and data - UK wont keep pace with europe; snoopers charter more likely to be passed with implications for data surveillance. |
| 6.30 | Less legislation may lead to less need to comply |
|  | Sample quotes – minimizing legal threats |
| 6.31 | make sure we know what is going on! |
| 6.32 | Implement GDPR entirely |
| 6.33 | keep ahead of current thinking and interpretation of dp adn confidentiality guidance and case law |
| 6.34 | Bi-lateral agreements post-Brexit |
| 6.35 | Follow best practices and regulations developed on an international level |
| 6.36 | Lobby government to keep existing legislation |
| 6.37 | Properly consider what works and what is unnecessary and bureacratic |
| 6.38 | Map laws to both digital need and citizen need |
| 6.39 | GDPR |
| 6.40 | stay in the EU!!! |
| 7.0 | ETHICAL FACTORS |
|  | Sample quotes – ethical opportunities |
| 7.1 | more and more organisations start to act upon identified hate texts of their employees against others. I think more sophisticated software could support this - maybe, by already trying to prevent it. E.g., indicating to the post writer that their post could be classified as hate speech and WHY it is not appropriate in the 21st century in countries with high HDI |
| 7.2 | Possibly chance for UK to rewrite ethical frameworks for IM |
| 7.3 | There is an opportunity to work with countries who have dubious ethics and to help them improve their ethical standards to enable them to trade with the UK |
| 7.4 | To be transparent, positive, inclusive, outward looking, and professional serving the needs of our students (and colleagues). |
| 7.5 | none |
| 7.6 | Stronger more democratic processes linked to transparent audited data and standards. |
| 7.7 | Professional ICT ethics crossing all info domains and bringing together global best practices. |
| 7.8 | Better leadership which is actually ethical not self serving |
| 7.9 | None foreseen |
| 7.10 | Keep EU laws |
|  | Sample quotes – harnessing ethical opportunities |
| 7.11 | Keep involved. Keep publishing. Develop and maintain international professional relationships |
| 7.12 | No more tax scandals from Brussels; UK taxation reporting will apply. |
| 7.13 | By campaiging to retain rights over information which are supported by EU directives or other legislation |
| 7.14 | In myriad ways |
| 7.15 | Media stop to report honestly what really happens |
| 7.16 | Teach the public information literacy skills and social ethical values! |
| 7.17 | Stay in the EU |
| 7.18 | To realise we need to live in a tolerant, open society where everyone ought to be given the right to prosper. |
| 7.19 | Redesign of policy to create greater accountability |
| 7.20 | Open government – with assurance that we all know it really is open! |
|  | Sample quotes – ethical threats |
| 7.21 | Free for all market dominance leading to a greater and greater acceptance of corruption and unethical practices |
| 7.22 | Theresa May as a leader is unethical in my view, so I can't her making ethical changes to policies. Brexit has threatened the whole idea of freedom of movement and international cooperation. It's a grim time for politics and the European community |
| 7.23 | Xenophobia; employment policies that deport existing (non-UK) EU nationals; discrimination against non-UK citizens; removal of legal and employment rights for non-UK employees |
| 7.24 | Lack of accountability and transparency |
| 7.25 | All protection of the above currently under the EU will need to be renegotiated and I do not have any confidence in the UK government that it will argue for any protection |
| 7.26 | NONE |
| 7.27 | Brexit seems to have given people license to be openly racist. Feels like we have gone backward as a society |
| 7.28 | Will overseas students come here to train on UK courses. Smaller pool of job applicants to choose from |
| 7.29 | Rise of the far right curtails expression and civil liberties |
| 7.30 | rise in rasism |
|  | Sample quotes – minimizing ethical threats |
| 7.31 | Pressure on government not to trigger article 50 |
| 7.32 | Fully endorse the EU freedom of people |
| 7.33 | Push for social justice agendas at an international level |
| 7.34 | Brexit |
| 7.35 | By campaigining |
| 7.36 | Ensure that the same restraints are put in place, whether it's by a new constitution or by maintaining the existing harmonised laws. |
| 7.37 | Petitioning the government to make ethical policies in the light of Brexit. |
| 7.38 | Transparent data |
| 7.39 | Open government as a de facto standard would help global government generally |
| 7.40 | Don’t leave the EU |
| 8.0 | Sample quotes – other comments |
| 8.1 | It is a golden opportunity for Britain to be Britain and every area of life will benefit. |
| 8.2 | The practical aspects of "replacing" the EU with the Commonwealth have not been explored in a pragmatic way. They should be. |
| 8.3 | Politically this will take time to resolve and my customers are already suggesting they have a preference for Non-UK teams due to uncertainty.  For European contracts we have already started to offer EU personnel where-as previously UK workers was a valued commodity. |
| 8.4 | I think information professionals and professional bodies are going to have to stay close to the process as Brexit unfolds over the coming years, to ensure that rights to information and access to archives and records are protected and that the cultural value of archives to the UK is demonstrated. |
| 8.5 | My library in Germany would like to continue it's collaboration with British partner libraries in EU-funded projects. This collaboration is under threat. |
| 8.6 | BREXIT is an excellent opportunity to engage more globally with democratic agendas |
| 8.7 | The Brexit is a disaster at all levels. The UK will shrink and become a narrow-minded isolated island missing out on all opportunities. |
| 8.8 | I think it is a terrible day for all UK citizens and other EU citizens. Brexit means the erosion of employment protection, environmental protection, economic hardship for all UK citizens, a weaker Europe. In addition, there has been a move to the right, including elements of the far right. I believe that library/information staff are going to be easy targets for job cuts. I do not have a positive outlook on any aspect of Brexit. |
| 8.9 | I think that Brexit was a huge step backwards for the UK. Back to closed borders and a hostile atmosphere of isolation and  nationalism. Plus, there might be economic consequences that can not be overcome. Drawbacks for workers, professionals or even tourists from outside the UK might be huge and cause people to avoid the UK in general. |
| 8.10 | Within broadcasting, I find it difficult to see that there will be much change. |
